# Contrasting patterns of neutral and functional genetic diversity in stable and disturbed environments

**DOI:** 10.1002/ece3.4667

**Published:** 2018-11-11

**Authors:** Yeşerin Yıldırım, Jon Tinnert, Anders Forsman

**Affiliations:** ^1^ Ecology and Evolution in Microbial Model Systems EEMIS Department of Biology and Environmental Science Linnaeus University Kalmar Sweden

**Keywords:** color polymorphism, dispersal, evolution, grasshoppers, population genetics

## Abstract

Genetic structure among and diversity within natural populations is influenced by a combination of ecological and evolutionary processes. These processes can differently influence neutral and functional genetic diversity and also vary according to environmental settings. To investigate the roles of interacting processes as drivers of population‐level genetic diversity in the wild, we compared neutral and functional structure and diversity between 20 *Tetrix undulata* pygmy grasshopper populations in disturbed and stable habitats. Genetic differentiation was evident among the different populations, but there was no genetic separation between stable and disturbed environments. The incidence of long‐winged phenotypes was higher in disturbed habitats, indicating that these populations were recently established by flight‐capable colonizers. Color morph diversity and dispersion of outlier genetic diversity, estimated using AFLP markers, were higher in disturbed than in stable environments, likely reflecting that color polymorphism and variation in other functionally important traits increase establishment success. Neutral genetic diversity estimated using AFLP markers was lower in disturbed habitats, indicating stronger eroding effects on neutral diversity of genetic drift associated with founding events in disturbed compared to stable habitats. Functional diversity and neutral diversity were negatively correlated across populations, highlighting the utility of outlier loci in genetics studies and reinforcing that estimates of genetic diversity based on neutral markers do not infer evolutionary potential and the ability of populations and species to cope with environmental change.

## 1 INTRODUCTION

Spatial and temporal changes in the environment can impact the size, stability, and connectedness of populations and thereby affect ecological processes such as dispersal, founder events, and extinction. In combination with selection, this molds functional genetic structure and diversity of natural populations. Neutral genetic variation is shaped by historic relationships, random processes such as genetic drift, recombination, and mutations, and gene flow between populations (Charlesworth & Charlesworth, 2017; Frankham, 1996; Reed & Frankham, 2001; Slatkin, 1987, 2008). Population dynamics are important in this context because small and fluctuating population sizes tend to reduce neutral genetic diversity within populations and can contribute to differentiation in neutral (but not necessarily functional) diversity among populations (Frankham, 1996; Kimura, 1983).

Adaptive or functional genetic diversity is influenced by the same suite of processes discussed above for neutral diversity. In addition, functional diversity is affected by differential fitness among individuals within populations (Endler, 1986; Holderegger, Kamm, & Gugerli, 2006) and by divergent selection in populations that inhabit different environments, potentially leading to local adaptations and differentiation (Dudaniec, Yong, Lancaster, Svensson, & Hansson, 2018; Johansson, Quintela, & Laurila, 2016; Lenormand, 2002; Noguerales, García‐Navas, Cordero, & Ortego, 2016; Quintela, Johansson, Kristjansson, Barreiro, & Laurila, 2014; Zhi‐Xiang, Fang, & Guo‐Fang, 2018). Unlike neutral diversity, functional genetic and phenotypic variability can in turn have a positive impact on the fitness of populations, by increasing evolvability, dampening fluctuations, increasing production of dispersers (emigrants), improving establishment success, and reducing extinction (Forsman, 2014; Forsman, Ahnesjö, Caesar, & Karlsson, 2008; Forsman & Wennersten, 2016; Forsman, Wennersten, Karlsson, & Caesar, 2012; Hughes, Inouye, Johnson, Underwood, & Vellend, 2008; Mills et al., 2018; Reed & Frankham, 2003; Rius & Darling, 2014; Vergeer, Sonderen, & Ouborg, 2004; Wennersten & Forsman, 2012; Whitlock, 2014; Willi, Van Buskirk, & Hoffmann, 2006).

Researchers are increasingly aware that the consequences and benefits associated with high intraspecific diversity can in turn profoundly influence processes at higher levels of organization (Bolnick et al., 2011; Des Roches et al., 2018; Whitlock, 2014). A recent meta‐analysis (Des Roches et al., 2018) indicates that, in aquatic systems, effects of intraspecific variation on ecological processes and ecosystem services are often comparable to, and sometimes stronger than, effects of species richness and species composition. This upward cascading includes effects of intraspecific diversity on community species composition, in part mediated by consequences of species traits, species interactions, and species sorting. An increased understanding of the causes and consequences of intraspecific diversity in a spatiotemporally changing world is thus essential for several areas in ecology and evolution.

**Figure 1:**
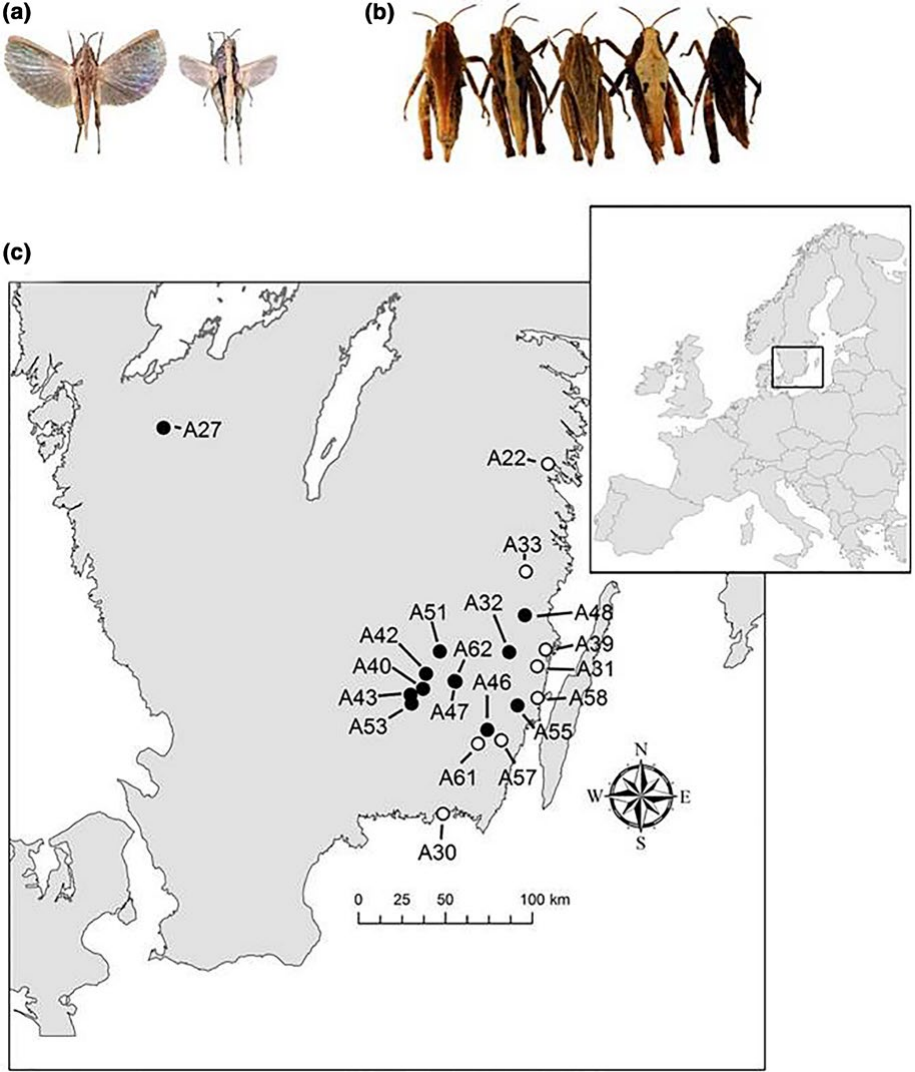
Wing morphs, color morphs, and study area. (a) *Tetrix undulata* pygmy grasshopper female belonging to the macropterous morph with long and functional wings (left) and micropterous short‐winged flightless morph (right). Photograph: J. Tinnert. (b) *Tetrix undulata* representing reddish brown, striped, striated brown, gray, and black color morphs. Photograph: A. Forsman. (c) Map of study area in the south of Sweden, showing location and Sample ID of 20 *Tetrix undulata* pygmy grasshopper populations in habitats representing stable (open circles) and disturbed (filled dots) environments. See Table 1 for a key to abbreviations of sampling locations

In the present study, we investigate the roles of ecological and evolutionary processes for population genetic structure and as drivers of phenotypic, functional, and neutral genetic diversity, and examine whether and how the relative importance of different processes change depending on ecological settings and conditions. To that end, we use data for populations of dispersal and color polymorphic pygmy grasshoppers (*Tetrix undulata* (Sowerby, 1806) Orthoptera, Tetrigidae) in disturbed and stable habitats (Figure 1).

*Tetrix undulata* is a small, diurnal, ground‐dwelling, and widely distributed insect that inhabits biomes ranging from the Mediterranean to arctic regions of Europe (Holst, 1986). It usually occupies drier microhabitats in relatively open areas where it lives on the soil surface and feeds on microalgae growing on moist soils, mosses, and detritus (Holst, 1986). Adult and late instar nymphs hibernate during winter and emerge in April–May when reproduction ensues in our study area (Figure 1). Females survive at most one reproductive season, produce multiple pods of egg (<35 eggs/clutch), and nymphs develop through five (males) or six (females) instars before eclosing. Because of its high reproductive capacity, *T. undulata* may rapidly become very numerous when conditions are favorable (Forsman, 2018).

Like many other insects, pygmy grasshoppers display dispersal polymorphism; a short‐winged flight‐incapable morph that lacks flight muscles coexists with a long‐winged flight‐capable morph (Berggren, Tinnert, & Forsman, 2012; Forsman, 2018; Harrison, 1980; Tinnert & Forsman, 2017) (Figure 1a). The discrepancy in dispersal capability among wing morphs generates a dynamic process, and a high initial incidence of long‐winged individuals in recently founded populations is followed by a decline in frequency over time (Berggren et al., 2012; Forsman, 2018). Populations in disturbed environments are therefore expected to show a high proportion of long‐winged phenotypes (Berggren et al., 2012; Denno et al., 1996) combined with low neutral genetic variation due to drift.

Pygmy grasshoppers provide a classic example of color polymorphism (Fisher, 1939; Forsman, 2018; Forsman, Karlsson, Wennersten, Johansson, & Karpestam, 2011; Nabours, 1929) (Figure 1b). The color polymorphism in pygmy grasshoppers provides a reliable proxy of functionally important genetic and phenotypic diversity within populations. Color variants of *T. undulata* differ in heat balance, thermal physiology, body size, reproductive life history, predator avoidance behaviors, microhabitat utilization, and diet, such that they occupy different niches and can be considered eco‐morphs (Ahnesjö & Forsman, 2003, 2006; Forsman, 2018; Forsman, Ahnesjö, & Caesar, 2007; Forsman, Ringblom, Civantos, & Ahnesjö, 2002). Color pattern affects the susceptibility to predation, and relative crypsis of morphs depends on the visual properties of their habitats (Forsman & Appelqvist, 1999; Karpestam, Merilaita, & Forsman, 2013; Tsurui, Honma, & Nishida, 2010). There is experimental evidence from pygmy grasshoppers that higher levels of color polymorphism protect individuals and populations against predation (Karpestam, Merilaita, & Forsman, 2016), reduce adverse effects of intraspecific competition (Caesar, Karlsson, & Forsman, 2010), and promote establishment success of founder groups (Forsman et al., 2012; Wennersten, Johansson, Karpestam, & Forsman, 2012). There is ample evidence that pygmy grasshopper color morphs are genetically influenced and not affected to any important degree by developmental plasticity (see Forsman, Karlsson et al., 2011; Forsman, 2018, references therein and Methods below).

Here, we use amplified fragment length polymorphism (AFLP) data (Bensch & Åkesson, 2005; Vos et al., 1995) to investigate genetic structure and diversity in 20 *T. undulata* populations in southern Sweden (Figure 1). Next, we compare wing morph frequencies, functional phenotypic variability as estimated using data on color morph diversity, and neutral and functional genetic variability based on AFLP loci between populations in disturbed and stable habitats.

We hypothesized that disturbed environments inhabited by recently established populations should be characterized by a high incidence of dispersal phenotypes; high diversity in functional traits because of the enhancing effect of diversity on establishment and persistence (Forsman, 2014, 2016; Forsman, Betzholtz, & Franzén, 2016; Forsman & Wennersten, 2016; Mills et al., 2018); and low neutral genetic diversity owing to the eroding effect of drift associated with small founder groups and fluctuating population sizes (Charlesworth & Charlesworth, 2017). The eroding effect of founder events is expected to leave a stronger signature on neutral than on functional genetic diversity in disturbed environments in part because establishment will typically fail if the group of colonizing individuals is not genetically diverse for functional traits (Forsman et al., 2012; Wennersten et al., 2012). By contrast, we hypothesized that populations in stable environments should be characterized by a lower incidence of dispersal phenotypes, unless much influenced by recent immigration (Harrison, 1980; Roff & Fairbairn, 2007); lower variability in functionally important traits, owing to the variance‐reducing effect of stabilizing selection (Arnold & Wade, 1984; Endler, 1986); and higher neutral genetic diversity, under the assumption that populations fluctuate less and that the eroding effect of founder events and bottlenecks therefore is weaker in stable environments (Charlesworth & Charlesworth, 2017).

## 2 MATERIALS AND METHODS

### 2.1 Sampling and study areas in contrasting environments

All applicable institutional and/or national guidelines for the care and use of animals were followed. We selected 20 sampling localities so that our dataset contained *T. undulata* populations with varying degrees of interpopulation distance and habitat stability. Sampling localities represented either undisturbed, relatively stable (*n *=* *8) habitats or disturbed (*n *=* *12) habitats (Figure 1, Table 1). Habitats in stable environments included stream shorelines, pastures, meadows, and agricultural land, all of which are influenced by small‐scale substrate surface modifications through trampling by farm animals, wave action, grazing by cattle and wild birds (e.g., geese and ducks), and agricultural activities. The modifications of soil surface and ground vegetation that result from these activities favor pygmy grasshopper behaviors, such as feeding and egg‐laying (Forsman, 2018). In contrast, sampling localities in disturbed environments represented habitats affected by more drastic and larger scale environmental makeovers associated with previous clear cuttings, natural forest fires, or managed fires. These makeovers can lead to extinction of local populations (if any) and profoundly change overall habitat structure, species community composition of plants and animals, and abiotic conditions including wind and sun exposure, surface temperatures, and humidity regimes. The conversion of forests, which are largely unsuitable as pygmy grasshopper habitats, through fires and cutting to open land with patches of bare soil and relatively few enemies, provides new habitats available for colonization, predominantly by long‐winged flight‐capable phenotypes. Overall, some species of pygmy grasshoppers seem well adapted to using a tracking strategy suitable for spatiotemporally changing environments and can thrive under these conditions (Forsman, 2018).

**Table 1:** Diversity in samples of *Tetrix undulata* pygmy grasshoppers collected from 20 sampling locations in stable and disturbed environments in the southeast of Sweden. *N* indicates number of individuals used for AFLP analyses, census population size is calculated based on information on number of collected individuals and capture probability, color morph unalikeability indicates color morph diversity estimated in a sample of (*N*) individuals, long‐winged indicate the proportion phenotypes with functional wings in a sample of (*N*) adult individuals, PL indicates number of polymorphic loci, PPL indicates percentage polymorphic loci, and Hj, nHj neutral, and sHj outlier indicate genetic diversity estimated using all 1,419 AFLP loci, 1,208 neutral AFLP loci, and 28 outlier AFLP loci, respectively. Distance to centroid indicates within‐group genetic dispersion as estimated using PERMDISP

Sample ID	Location	Year	*N*	Habitat type	Disturbance regime	Census pop. size	Color morph unalikeability (*N*)	Long winged (*N*)	Coordinates Lat. Long.	Elevation (m.a.s.l.)	PL	PPL	Hj all (*SE*)	nHj neutral	sHj outlier	Dist. to centroid neutral	Dist to centroid outlier
A22	Tindered	2012	11	Pasture	Stable	40	0.68 (16)	0.13 (15)	57.977083°, 16.484550°	25	1136	80.1	0.294 (0.00486)	0.318	0.357	0.368	0.266
A30	Tomtesunda	2012	17	Pasture	Stable	42	0.73 (17)	0 (17)	56.173883°, 15.479167°	14	1011	71.2	0.270 (0.00481)	0.293	0.422	0.322	0.245
A31	Ålem	2009	6	Agricultural land	Stable	180	0.88 (26)	0.04 (26)	56.933650°, 16.363467°	23	968	68.2	0.271 (0.00489)	0.291	0.475	0.260	0.219
A33	Hägern	2009	9	Meadow nearby burnt area	Stable	98	0.85 (28)	0.04 (28)	57.423067°, 16.266067°	62	1012	71.3	0.262 (0.00492)	0.284	0.421	0.298	0.262
A39	Oknebäck	2009	15	Stream shoreline	Stable	110	0.89 (41)	0.17 (41)	57.019533°, 16.444967°	4	946	66.7	0.253 (0.00481)	0.275	0.374	0.307	0.267
A57	Aspelund	2008	14	Pasture with stream	Stable	102	0.77 (18)	0 (18)	56.553767°, 16.022617°	46	1171	82.5	0.294 (0.00483)	0.323	0.310	0.379	0.336
A58	Björnö	2008	10	Pasture	Stable	65	0.86 (13)	0 (5)	56.770617°, 16.364550°	4	1124	79.2	0.289 (0.00470)	0.317	0.336	0.286	0.371
A61	Sävsjö	2008	5	Pasture with pond	Stable	95	0.69 (7)	0 (7)	56.537800°, 15.803867°	110	1105	77.9	0.347 (0.00438)	0.376	0.296	0.380	0.366
A27	Fåglum	2012	15	Clear cut with pond	Disturbed	38	0.72 (15)	0 (4)	58.128183°, 12.733633°	108	859	60.5	0.230 (0.00497)	0.251	0.334	0.290	0.289
A32	Flyvägen	2009	23	Burnt clear cut	Disturbed	345	0.88 (136)	0.44 (135)	57.007950°, 16.100367°	82	1105	77.9	0.285 (0.00475)	0.307	0.482	0.334	0.293
A40	Lessebo hygge	2010	28	Clear cut	Disturbed	138	0.89 (55)	0.36 (55)	56.735017°, 15.285967°	179	997	70.3	0.251 (0.00474)	0.274	0.348	0.319	0.277
A42	Nässjön	2008	24	Managed fire	Disturbed	70	0.85 (27)	1 (1)	56.895650°, 15.312917°	238	925	65.2	0.238 (0.00476)	0.258	0.335	0.319	0.279
A43	Hovmantorp	2010	25	Burnt clear cut	Disturbed	348	0.83 (139)	0.74 (135)	56.786150°, 15.167550°	172	1042	73.4	0.276 (0.00466)	0.299	0.421	0.317	0.311
A46	Påryd	2012	24	Burnt area	Disturbed	125	0.92 (50)	0.26 (50)	56.608017°, 15.892667°	100	880	62.0	0.238 (0.00501)	0.256	0.370	0.314	0.387
A47	Filmstället	2006	12	Burnt clear cut	Disturbed	272	0.87 (109)	0.79 (109)	56.856150°, 15.594033°	217	1092	77.0	0.261 (0.00478)	0.285	0.261	0.302	0.394
A48	Fliseryd	2007	18	Burnt area	Disturbed	75	0.91 (30)	1 (30)	57.197717°, 16.250350°	60	968	68.2	0.246 (0.00488)	0.270	0.254	0.311	0.351
A51	Sävsjöström	2004	20	Burnt area	Disturbed	778	0.88 (311)	0.73 (241)	57.011300°, 15.443083°	231	1056	74.4	0.271 (0.00474)	0.294	0.333	0.335	0.421
A53	Linneryd	2007	22	Clear cut	Disturbed	75	0.85 (30)	0.93 (28)	56.741600°, 15.181133°	161	1034	72.9	0.261 (0.00485)	0.285	0.277	0.332	0.413
A55	Läckeby	2007	21	Clear cut	Disturbed	105	0.85 (42)	0.08 (40)	56.732550°, 16.175767°	36	1022	72.0	0.261 (0.00486)	0.285	0.261	0.329	0.418
A62	Gölen	2006	16	Burnt clear cut	Disturbed	170	0.84 (68)	0.71 (52)	56.858667°, 15.580783°	216	981	69.1	0.270 (0.00490)	0.298	0.245	0.348	0.289
	Total		335														

Disturbed sites were located, on average, at greater distances from the coast (Figure 1) and at higher elevation (mean: 150, range: 36–238 m above sea level) compared with stable (mean: 36, range: 4–110) sites (Table 1). It might therefore be hypothesized that some other environmental factor(s) not related to disturbance have influenced the genetic differentiation and diversity of the populations under study. However, our analyses suggest that this is unlikely (see Results).

Grasshoppers were collected in spring and early summer (for details, see Forsman, Karlsson et al., 2011, 2012; Tinnert, Hellgren, Lindberg, Koch‐Schmidt, & Forsman, 2016; Tinnert & Forsman, 2017). Individuals were identified to species according to Holst (1986), classified according to their sex, wing morph (long‐winged with functional wings or short‐winged and flightless) (Berggren et al., 2012; Tinnert & Forsman, 2017), and color morph. All or a subset (depending on how many were collected) of the collected individuals were preserved in 90% ethanol until DNA extraction (see below). The number of individuals used for AFLP analyses is typically different from the sample sizes used to calculate the proportion of long‐winged individuals and color morph diversity (Table 1). These discrepancies arose because some of the collected individuals were nymphs, which can be used for AFLP analyses but not for classification of wing or color morph. For some sampling locations, only a subsample of the collected individuals was brought to the laboratory for classification of wing morph, color morph, and DNA extraction, whereas remaining individuals were released at the sampling location.

### 2.2 Estimating population size and immigration rate

Census population size (Table 1) was estimated for each locality based on information on number of collected individuals adjusted for capture probability (Tinnert & Forsman, 2017), which was set to 0.4 based on previous extensive capture–mark–recapture studies (Berggren et al., 2012; Forsman & Appelqvist, 1999; Tinnert & Forsman, 2017; Tinnert, Hellgren et al., 2016). We searched for grasshoppers while walking slowly through the area during days with weather conditions suitable for grasshopper activity, that is, clear or overcast days with a temperature of at least 15°C (Forsman, Karlsson et al., 2011, 2012). The number of individuals collected at each site underestimates actual population size, but it is a reliable relative measure which is robust to factors such as differences in the area covered for sampling, time invested in sampling, number of people involved in each sampling event, habitat type, and weather conditions that could potentially influence total catch (Tinnert & Forsman, 2017; Tinnert, Hellgren et al., 2016).

The proportion of long‐winged phenotypes (Figure 1a) in each population was used as a proxy for recent colonization or immigration events (Table 1). Previous studies show that the proportion of pygmy grasshopper males and females that belong to the macropterous long‐winged morph is highly correlated across samples from different populations and years, that the incidence of the long‐winged morph does not differ consistently between males and females (Berggren et al., 2012; Forsman, 2018; Tinnert, Hellgren et al., 2016), and that capture probability is independent of both sex (Forsman & Appelqvist, 1999) and wing morph (Berggren et al., 2012). It is therefore unlikely that the estimates of the proportion of long‐winged phenotypes reported in Table 1 were influenced to any important degree by sampling bias according to sex or wing morph, or by any differences in sex ratio among samples from different collection sites. Furthermore, only the macropterous phenotype is able to fly (Berggren et al., 2012). Collectively, this suggests that a high incidence of the long‐winged flight‐capable morph may be used as a proxy to identify *T. undulata* populations that have been recently established or that represent older populations that have been much influenced by recent immigration (and that might show signatures associated with admixture) (Tinnert, Berggren, & Forsman, 2016; Tinnert & Forsman, 2017; Tinnert, Hellgren et al., 2016). Available evidence indicates that wing morph in pygmy grasshoppers is heritable and not influenced to any important degree by developmental plasticity (Berggren et al., 2012; Forsman, 2018). Available evidence also indicates that wing morph is independent of color morph (Forsman, 2018; Forsman, Karlsson et al., 2011).

### 2.3 Estimating color morph diversity

Pygmy grasshopper color morphs range from light gray via different shades of brown to black, some being uniform and others mottled or patterned with longitudinal stripes, vertical bars, or speckles (Ahnesjö & Forsman, 2003, 2006; Forsman, 2018; Forsman et al., 2002, 2007) (Figure 1b). Individuals vary also with regard to texture of the integument, the surface being either smooth, granular or consisting of longitudinal ridges and grooves. Split‐brood experiments have shown that neither the patterning nor the overall darkness of pattern elements is influenced by substrate, temperature, or crowding (Ahnesjö & Forsman, 2003; Forsman, 2011; Karlsson & Forsman, 2010; Karlsson, Johansson, Caesar, & Forsman, 2009). Further, color morph frequencies in samples of wild‐caught individuals from different populations are highly correlated with those in captive‐reared individuals, indicative of population‐level heritability (Forsman, Karlsson et al., 2011), and there exists as yet no firm evidence that the color polymorphism in pygmy grasshoppers is affected by developmental plasticity (for a detailed discussion, see Karlsson et al., 2009).

To quantify color morph diversity in populations, we used the coefficient of unalikeability (Kader & Perry, 2007; Perry & Kader, 2005). Measures of population variability for categorical characters are different from those used for quantitative or continuous traits where variation about the mean is the norm. The coefficient of unalikeability (*u*_2_) is a measure of the proportion of possible comparisons which are unalike and was calculated based on the equation: $u_{2}=1-\sum_{i=1}p_{i}^{2}$, where *p* represents the proportion of the population in each of the *i* ^th^ categories of the categorical variable (i.e., color morph) (Kader & Perry, 2007; Perry & Kader, 2005). Unalikeability can take any value from 0.0 to 1.0, with higher values representing higher diversity. Estimates of color morph unalikeability for our study populations were independent of sample size (*r *=* *0.35, *n *=* *20, *p *=* *0.14).

### 2.4 DNA extraction and molecular genetics analyses

From each of the 20 sampling locations, we used 5–28 individuals for DNA extraction and genetic analysis (Table 1). DNA was extracted from the femur of each individual using phenol–chloroform method according to Sambrook (Sambrook, Fritch, & Maniatis, 2002) (see Supporting Information for details).

Analysis of AFLP was carried out as described previously (Bensch & Åkesson, 2005; Vos et al., 1995), using the restriction enzymes EcoRI and Tru1 pre‐amplification primers M_A_ X E_A_ and combinations of four selective primers (pair 1—E_TAG_ X M_CGA_, pair 2—E_TAG_ X M_CAG_, pair 3—E_TCG_ X M_CAC_, and pair 4—E_TAG_ X M_CAC_) (Tinnert & Forsman, 2017; Tinnert, Hellgren et al., 2016). Three negative and nine positive controls were included on each plate. The fragment analyses were performed by Uppsala Genome Center with an ABI3730XL DNA Analyzer (Applied Biosystems, USA). Resulting chromatograms were analyzed in GeneMapper 5.0 (Applied Biosystems) for allele calling (see Supporting Information for details). A total of 1,419 polymorphic sites were used for further analysis. Peak heights were normalized using AFLPscore (Whitlock, Hipperson, Mannarelli, Butlin, & Burke, 2008) and were scored as present if the peaks were >15% of the mean peak height of a particular locus. We obtained a resulting binary matrix, consisting of ones (presence) and zeros (absence of the fragment).

### 2.5 Detection of loci under selection

We used a Bayesian approach implemented in BayeScan v.2.1 (Foll & Gaggiotti, 2008) to detect the outlier loci. The analysis was performed with 20 pilot runs and a 200,000 step burn‐in followed by 200,000 iterations (a sample size of 5,000 and thinning interval of 20). Posterior odds (PO; the ratio of posterior probabilities of selection over neutrality) ratio was set to 100. Low polymorphic loci with dominant allele frequency <2% and >98% across the whole dataset were removed to minimize false discovery rate. The original dataset was divided into two subsets based on the results of the BayeScan: outlier loci under selection (sDATA) and neutral loci (nDATA). Loci with log_10_ PO > 2 (decisive evidence for selection) were regarded as the outliers (loci under selection). To obtain a neutral dataset, loci with log_10_ PO > 1 (strong evidence, Jeffrey, 1961) were removed from the dataset to exclude also potential outliers.

### 2.6 Estimating genetic diversity within populations

Overall *F*_ST_ and genetic diversity indices were calculated in AFLPsurv v1.0 (Vekemans, 2002) for all loci, all neutral loci (nDATA), and all outlier markers (sDATA). We used a Bayesian method with nonuniform prior distribution of allele frequencies (Zhivotovsky, 1999). We assumed Hardy–Weinberg equilibrium, as *T. undulata* is neither highly self‐fertilizing nor haploid. Additionally, preliminary inbreeding coefficient (*Fis*) estimations for a subset of eight populations suggested a range of *Fis* values (0.06 to 0.68) randomly distributed among the populations without suggesting a plausible *Fis* > 0 values for all populations. These calculations were performed in I4A (Chybicki, Oleksa, & Burczyk, 2011) with default settings, except that 50,000 sampling steps were used. Statistics of genetic diversity included number of polymorphic loci (PL), proportion of polymorphic loci (PPL) at the 5% level, and Nei's genetic diversity (Hj) (Nei, 1973) for both neutral (nHj) and outlier (oHj) loci. In addition to estimating within‐population genetic diversity using Hj, PERMDISP tests performed with PRIMER v.7 (Clarke & Gorley, 2006) with PERMANOVA+ (Anderson, Gorley, & Clarke, 2008) were used to test the null hypothesis of homogeneity of within‐group dispersions among populations and between stable and disturbed environments (Anderson, 2006). PERMDISP uses principal coordinates generated from a Jaccard distance matrix (Bonin, Ehrich, & Manel, 2007) to compare the average deviations from centroids (Anderson, 2006), and we did this separately for neutral and outlier loci. If the distances of individuals from one population to its centroid are larger than distances to the corresponding centroid in another population, this suggests that the diversity is greater in the former group (Parker, Anderson, Jenkins, & Brunton, 2012).

PERMDISP is normally used primarily for checking the assumption of homogeneity of variance and determine whether differences in within‐group dispersion are likely to confound results and conclusions from PERMANOVA regarding variation in overall genetic structure among populations (Anderson, 2006; Anderson & Walsh, 2013). Here, in addition to that (see the next subsection), we used PERMDISP to test for a difference in the level of genetic diversity between populations in stable and disturbed environments.

### 2.7 Analyses of genetic structure among populations

It is possible that environmental factors associated with geographic location (coastal versus inland) or with differences in elevation have resulted in genetic differentiation among populations. It is also possible that the existence of disturbed and stable environments has favored the evolution of genetically different ecotypes of pygmy grasshoppers. To evaluate these hypotheses, population genetic structure was investigated using estimates of pairwise *F*_ST_ values (Weir & Cockerham, 1984) as implemented in ARLEQUIN v.3.5 (Excoffier & Lischer, 2010) using 10,000 permutations.

An analysis of molecular variance AMOVA (Excoffier, Smouse, & Quattro, 1992) was performed in ARLEQUIN to determine the hierarchical genetic structure based on *F*_ST_ for the nDATA and sDATA datasets. Two separate calculations were performed for each dataset: one with structure and one without structure. For the former, to partition the total genetic variation and test for overall differentiation between grasshopper populations in disturbed and stable environments and according to geographic location or elevation, we performed a nested AMOVA where the populations were nested in either of two disturbance regimes (disturbed or stable). For the latter, all 20 populations were grouped together. The statistical significance of the AMOVA was tested by 10,000 permutations.

In addition to AMOVA, a multivariate approach was used to investigate the effects of sampling location and environment (disturbed–stable) on the distribution of the genetic variation among *T. undulata* individuals. Jaccard distances between the individuals (Bonin et al., 2007), separately for neutral and outlier loci, were calculated in FAMD v1.3 (Schluter & Harris, 2006), and the resulting matrices were used for the further statistical analysis that was performed in PRIMER v7. The matrices were visualized using nonmetric multidimensional scaling ordination (MDS) (Kruskal, 1964). Permutational multivariate analysis of variance (PERMANOVA) (Anderson, 2001; McArdle & Anderson, 2001) was used to test for differences in genetic structures among different sampling locations (populations) and between stable and disturbed environments. The sampling locations were introduced as a random factor and disturbance regime as a fixed factor. All permutation tests used 9,999 unrestricted permutations and partial sums of squares type (Type III). A significance level of α ≤ 0.05 was accepted. Results from PERMDISP analyses indicated that there were differences in genetic dispersion between populations in disturbed and stable environments (see Results). Results from PERMANOVA pointing to any difference in overall genetic structure between environments should therefore be interpreted with caution (Anderson & Walsh, 2013).

To evaluate the isolation‐by‐distance hypothesis (IBD) (Slatkin, 1993; Wright, 1943), we estimated the correlation between matrices of genetic (as estimated by *F*_ST_) and geographic (log km) distance using a Mantel test (Excoffier & Lischer, 2010). Geographic pairwise distance was calculated from GPS coordinates in Geographic Distance Matrix Generator v1.2.3 (Ersts, 2012).

To examine whether genetic structure and pairwise divergence among populations in neutral AFLP loci were associated with the divergence in outlier AFLP loci putatively influenced by selection, we used a Mantel test. Mantel tests were performed in the zt software (Bonnet & Van de Peer, 2002) with 9,999 permutations.

### 2.8 Statistical analyses

Analyses of genetic structure uncovered significant differentiation among most study populations and no signature of isolation by distance (see Results), justifying that the different study populations can be considered as independent observations. We tested for differences between populations inhabiting disturbed and stable environments in proportion of long‐winged phenotypes, color morph diversity (coefficient of unalikeability), neutral and outlier genetic diversity, and census population size using either *t* tests or nonparametric Kruskal–Wallis tests, depending on whether data violated assumptions of homogeneity of variances or normality. We investigated whether genetic diversity as estimated based on neutral and outlier AFLP data was associated with the level of within‐population diversity in a functionally important phenotypic trait (color pattern) using correlation analysis. Prior to statistical analyses, the proportion of long‐winged individuals was arcsine‐square root‐transformed, and census population size was log10‐transformed.

## 3 RESULTS

### 3.1 Outlier loci identified using selection test

Of the 1,419 AFLP loci, 1,277 had dominant allele frequencies >2% and <98%, and they were included into the outlier analyses. According to BayeScan analysis, 28 (of the 1,277 = 2.2%) loci exceeded the threshold for “decisive” evidence of selection with a FDR of ≤0.001043 as the outliers, and they were used for the outlier dataset (sDATA). Alpha values were positive for all 28 loci, suggesting diversifying selection. In addition to these 28 loci, 41 loci that exceeded the threshold of “strong” evidence of selection were excluded from the neutral dataset (nDATA) that included 1,208 loci. These 41 loci also had positive alpha values, except one with negative value suggesting balancing or purifying selection.

### 3.2 Population genetic variance within populations

Analyses of the 1,419 polymorphic AFLP markers (the dataset ALL) revealed high within‐population genetic diversity estimated with proportion polymorphic loci (PPL) and average heterozygosity (Nei's genetic diversity, Hj) across all sampling localities (PPL = 60.5 to 82.5; Hj = 0.23 to 0.35, Table 1). Hj and PPL were correlated across sampling locations (*r *=* *0.84, *n *=* *20, *p *<* *0.0001). Estimates of Nei's genetic diversity and of mean deviations from centroid for neutral (DevCneu) and for outlier loci (DevCout) are reported for each population in Table 1. Estimates of neutral AFLP diversity within populations based on nHj were positively correlated with estimates based on deviations from centroid (*r *=* *0.60, *p *<* *0.0049, *n *=* *20). Estimates of functional AFLP diversity within populations based on sHj and deviations from centroid of outlier loci (DevCout) were negatively correlated (*r *=* *−0.62, *p *=* *0.0038, *n *=* *20). Calculation of Hj is dependent on correct *Fis* values (Vekemans, 2002). Because we did not have a realistic *Fis* value applicable to all populations (see Methods, section ), it is possible that estimates of sHj might deviate from the actual values. By comparison, multivariate analyses (including PERMDISP) do not require a specific genetic model of populations, and we therefore consider the deviations from the centroid more reliable estimates for quantification and comparisons of within‐population diversity.

Estimates of neutral and functional genetic diversity were not strongly or consistently associated with sample sizes (the number of individuals from each site used for AFLP analyses, nHj: *r *=* *−0.19, *p *=* *0.42 sHj: *r *=* *−0.34, *p *=* *0.14; DevCneu *r *=* *0.13, *p *=* *0.57; DevCout *r *=* *0.28, *p *=* *0.24, all *n *=* *20).

### 3.3 Population genetic structure

Results of the nested AMOVA did not reveal any significant overall genetic differences depending on disturbance regime (Supporting Information Table S1), indicating that disturbed and stable environments were not populated by grasshoppers that represented different reproductively isolated evolutionary lineages. This lack of difference also demonstrates that populations that inhabited sites that were closer to the east coast and at lower elevation were not genetically separated or different overall from inland populations (Figure 1c). Sampling location accounted for 9% of the total genetic variation, and 91% was explained by variation among individuals within sampling locations (Supporting Information Table S1). Comparisons of results of outlier and neutral AFLP loci showed that sampling location accounted for a greater portion of the total variance in functional (32%) than in neutral (8%) genetic variation (Supporting Information Table S1).

**Figure 2:**
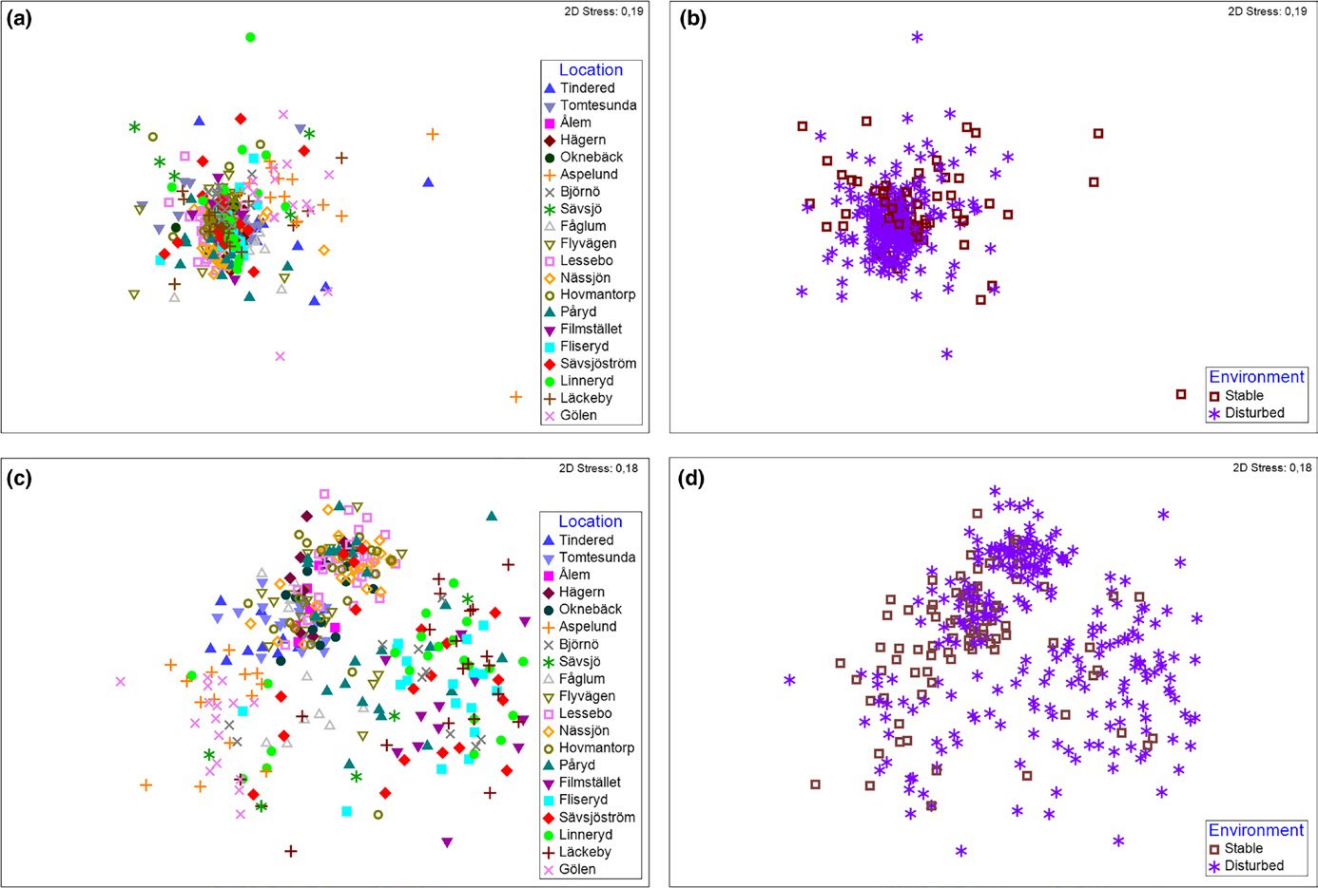
Multidimensional scaling ordination of Jaccard distances based on neutral (a,b) and outlier (c,d) AFLP loci visualized according to 20 sampling locations (a,c) and two environmental states (stable or disturbed, b,d)

Results from PERMANOVA were similar to those reported above for AMOVA, for both neutral and outlier loci. When populations were nested within environments (stable or disturbed), there was no significant signature of environment (neutral: *F*_1,315_ = 1.33, *p *=* *0.065; outlier: *F*_1,315_ = 1.60, *p *=* *0.14), whereas the location effects were strong (neutral: *F*_18,315_ = 3.00, *p *=* *0.0001; outlier: *F*_18,315_ = 16.99, *p *<* *0.0001). The lack of the genetic differentiation between different regimes is also evident in multidimensional scaling ordination plots based on analysis of Jaccard distances for neutral and for outlier loci according to population and environmental state (Figure 2).

Pairwise genetic differentiation among populations, as estimated by *F*_ST_, was generally lower for neutral than for outlier loci and ranged from 0 to 0.16 for neutral and from 0 to 0.58 for outlier loci (Supporting Information Table S2). Nearly all (174 and 175 of 190 for the neutral and outlier loci, respectively) of the pairwise comparisons were statistically significant after FDR correction (Benjamini & Hochberg, 1995) (Supporting Information Table S2, Figure 2). There was no clear signature of isolation by distance. Pairwise genetic differences, as estimated by *F*_ST_, were not correlated with the pairwise geographic distance separating sampling locations (Mantel test: neutral AFLP loci: *r *=* *0.248, *p *=* *0.099; outlier AFLP loci: *r *=* *0.045, *p *=* *0.280).

Analysis based on pairwise *F*_ST_ values suggested that the genetic divergence between populations in neutral AFLP loci was correlated with the genetic divergence in outlier AFLP loci (Mantel test, *r *=* *0.64, *p *<* *0.0001).

### 3.4 Comparisons of populations between contrasting disturbed and stable environments

**Figure 3:**
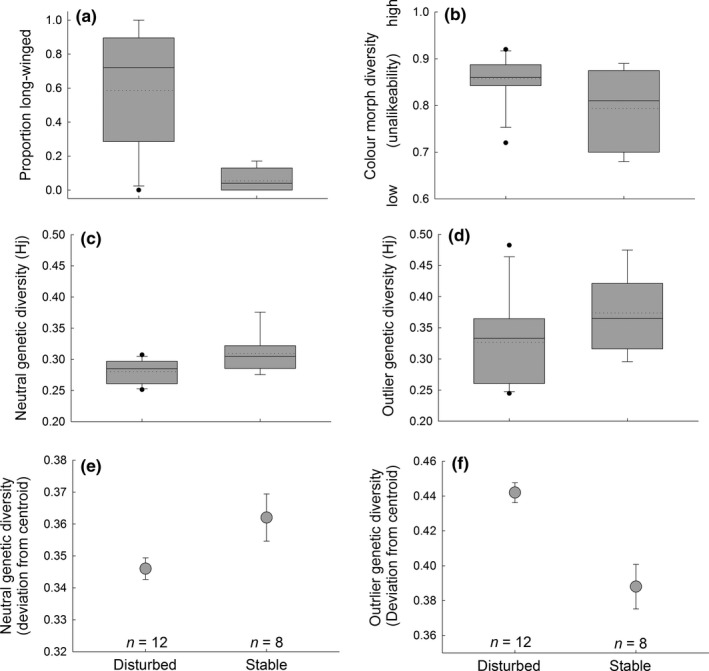
Results from contrasting environment comparisons. Comparisons of (a) immigration rate (as estimated using proportion long‐winged phenotypes), (b) functional color morph diversity (as estimated using unalikeability), (c) neutral genetic diversity (Hj, as estimated from AFLP data), (d) outlier genetic diversity (Hj, as estimated from AFLP data), (e) genetic dispersion of neutral ALFP loci (estimated using PERMDISP as deviations (mean ± *SE*) from centroid), and (f) genetic dispersion of outlier AFLP loci between 20 populations of *Tetrix undulata* pygmy grasshoppers collected from disturbed or from stable environments (see text and Table 1 for description of sampling locations and sample sizes). *N*‐values above horizontal axis in bottom panel indicate number of populations in each type of environment

The proportion of long‐winged phenotypes (indicative of recent establishment and immigration) was higher on average in populations in disturbed (mean = 0.59, range: 0–1.0) than in stable (0.05, 0–0.17) environments (Kruskal–Wallis χ^2^ = 9.68, *df* = 1, *p *=* *0.0019, Figure 3a).

Color morph diversity (a proxy of functional phenotypic and genetic diversity) was higher in disturbed (mean unalikeability ± *SD*, 0.86 ± 0.05) than in stable (0.79 ± 0.09) environments (Kruskal–Wallis χ^2^ = 2.08, *df* = 1, *p *=* *0.05, Figure 3b).

Neutral genetic diversity (estimated by nHj based on AFLP data after omitting outlier loci) was lower in disturbed (0.28 ± 0.018) than in stable (0.31 ± 0.032) environments (*t *=* *2.61, *df* = 18, *p *=* *0.0178, Kruskal–Wallis χ^2^ = 4.02, *df* = 1, *p *=* *0.045, Figure 3c).

Functional AFLP genetic diversity (estimated by sHj based on outlier AFLP loci) did not differ significantly between disturbed (0.33 ± 0.0731) and stable (0.37 ± 0.062) environments (*t *=* *1.49, *df* = 18, *p *=* *0.115, Figure 3d).

Results from PERMDISP analysis confirmed that dispersion (within‐group genetic variability estimated as deviation from centroid) in neutral loci was significantly lower in disturbed (mean deviation from centroid ± *SE*, 0.346 ± 0.0034) than in stable (0.362 ± 0.0074) environments (*F*_1,333_ = 5.18, *p *=* *0.032) (Figure 3e). However, for outlier loci, the pattern was opposite, with genetic dispersion being greater in disturbed (0.442 ± 0.0057) than in stable (0.388 ± 0.0128) environments (*F*_1,333_ = 19.29, *p *<* *0.0002) (Figure 3f).

There was no difference in census population size between populations in disturbed (mean = 211, range: 38–778 individuals) and stable (mean = 92, range: 40–180) environments (*t *=* *1.75, *df* = 18, *p *=* *0.098).

### 3.5 Associations of functional phenotypic diversity with genetic diversity, immigration, and census population size

Color morph diversity decreased with increasing neutral genetic diversity across all populations (deviation from centroid: *r *=* *−0.51, *n *=* *20, *p *=* *0.022) but was not associated with outlier genetic diversity (*r *=* *0.22, *n *=* *20, *p *=* *0.36).

Color morph diversity increased with increasing immigration rate (as estimated using proportion of long‐winged phenotypes, *r *=* *0.46, *n *=* *20, *p *=* *0.04).

Color morph diversity increased with increasing census population size (*r *=* *0.52, *n *=* *20, *p *=* *0.018).

## 4 DISCUSSION

By virtue of their dispersal polymorphism, color polymorphism, life history, and transient, habitat‐tracking, meta‐population like dynamics, the well‐studied pygmy grasshoppers lend themselves admirably for investigating drivers of population‐level genetic diversity (Forsman, 2018; Tinnert & Forsman, 2017). Overall, our present results illustrate how the genetic structure and diversity of natural populations are influenced by a combination of ecological and evolutionary processes, the relative importance of which differ for neutral and functional genetic diversity and vary according to ecological conditions, such as environmental disturbance regime.

Specifically, analyses based on both neutral and outlier AFLP markers revealed significant differentiation among most study populations (174 and 175 of 190 for the neutral and outlier loci, respectively). Results from comparisons between contrasting environments uncovered that populations in disturbed habitats had a higher incidence of long‐winged phenotypes, as expected if these populations were recently established by flight‐capable colonizers. Intrapopulation functional color morph diversity was also higher in disturbed than in stable environments. This outcome was expected given that color polymorphism increases establishment success (Forsman, 2014; Forsman et al., 2012; Wennersten et al., 2012). Neutral genetic diversity varied among populations, and, unlike functional diversity, it was lower (not higher) overall in disturbed than in stable habitats. This pattern indicates that the eroding effects on neutral diversity of genetic drift associated with recent founding events or population bottlenecks were stronger in disturbed compared to stable habitats. Finally, there was a statistically significant negative overall correlation between functional phenotypic and neutral genetic diversity across populations.

### 4.1 Contrasting environments—diversity in disturbed and stable habitats

**Figure 4:**
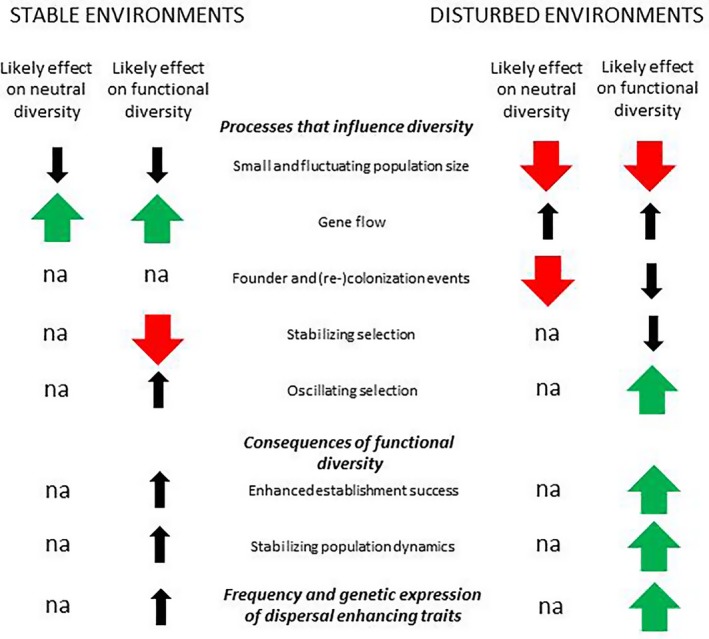
A schematic illustration and summary of how random and deterministic processes differently influence neutral and functional genetic diversity of populations in stable and disturbed environments. Green (up) and red (down) thick arrows refer to positive and negative effects, respectively, and indicate that the magnitude of the effect is greater than in the other environmental disturbance regime. Black thin arrows indicate that the effects might be present albeit of relatively small magnitude compared with those indicated by red and green thick arrows. na indicates not applicable

The pattern of genetic structure and intrapopulation diversity seen in these grasshoppers can be accounted for by combined effects of ecological and evolutionary processes associated with environmental change, as summarized in Figure 4 and discussed below.

According to literature, *T. undulata* is an almost exclusively short‐winged species (Holst, 1986). This is in concordance with the incidence of the long‐winged morph (0–17%) in populations in stable habitats. The higher proportion of the long‐winged dispersive phenotype in disturbed habitats (59% on average) suggests that these populations were founded by individuals carrying genes for long‐winged phenotypes shortly after the disturbance event (Berggren et al., 2012). The lack of association across populations of neutral genetic diversity with long‐winged phenotypes is also in agreement with the interpretation that the populations in disturbed environments represented new establishments, rather than older populations having been influenced by gene flow and admixture. If the founders of recently established populations had originated from different source populations, one would expect both functional and neutral genetic diversity to be high in disturbed environments (Hedrick, 2006; Simberloff, 2009). However, pygmy grasshopper populations can be established by very few individuals (Forsman et al., 2012; Wennersten et al., 2012), indicating that founder groups of divergent origins are not necessarily very common. Older populations that are much influenced by immigration might also display high frequencies of long‐winged individuals, but this should not be accompanied by low neutral diversity.

Color morph diversity and within‐group dispersion of outlier genetic diversity were higher in disturbed than in stable habitats. This is most likely a consequence of the higher survival and establishment success of more color morph diverse pygmy grasshopper founder groups (Forsman et al., 2012; Wennersten et al., 2012). The positive effects of functional diversity on colonization success, stability, and persistence of populations (Forsman, 2014, 2016; Forsman, Betzholtz, & Franzén, 2015; Forsman & Wennersten, 2016; Hughes et al., 2008; Karpestam et al., 2016) are probably sufficiently strong to outbalance the eroding effect of drift. Additionally, opposing selection in males and females (Forsman, 2018; Forsman & Appelqvist, 1999; Karpestam, Merilaita, & Forsman, 2014) together with the promiscuous mating behavior of pygmy grasshoppers (Caesar, Ahnesjö, & Forsman, 2007; Johansson, Caesar, & Forsman, 2013) may prevent the erosion of functional genetic diversity.

The higher color morph diversity and the higher genetic diversity of outlier AFLP loci in disturbed compared with stable environments may be attributable in part to different selection regimes. In disturbed environments, oscillating and temporally changing selection associated with succession and habitat modifications resulting in rapid temporal shifts in morph frequencies (Forsman, Karlsson et al., 2011; Karlsson, Caesar, Ahnesjö, & Forsman, 2008; Karpestam et al., 2013) may promote and preserve genetic variation. In stable environments, by contrast, stabilizing selection likely reduces genetic variance (Arnold & Wade, 1984; Endler, 1986) and favors the adaptation to optimum phenotypes (de Vladar & Barton, 2014).

The difference in neutral genetic diversity between populations in stable and disturbed environments was opposite to that seen in functional phenotypic diversity and in outlier AFLP loci. That neutral genetic diversity was lower in populations that occupied disturbed compared with stable habitats is in agreement with the interpretation that populations in disturbed habitats were the results of recent colonization events and affected by genetic bottlenecks that lowered primarily neutral genetic diversity. There is experimental evidence that pygmy grasshopper populations can be established by founder groups consisting of no more than six individuals (Forsman et al., 2012), thus leaving ample opportunity for eroding effects of founder events on neutral diversity. These eroding neutral processes were countered by other, deterministic and selective, processes that influenced and were influenced by functional (but not neutral) diversity, as discussed above.

Across populations, there was a negative correlation between color morph diversity and neutral AFLP diversity. However, color morph diversity was not associated with diversity in outlier AFLP loci. These findings are in agreement with predictions from theory and add to the body of evidence (Holderegger et al., 2006; Leinonen, O'Hara, Cano, & Merilä, 2008; Reed & Frankham, 2001; Whitlock, 2014; Willi et al., 2006) that estimates of neutral genetic diversity cannot be used as substitutes for adaptive or functional genetic diversity to infer evolutionary potential and the ability of populations and species to cope with environmental change.

One interpretation of the negative association between functional and neutral diversity would be that selection has been more efficient in removing unfit color morphs in larger populations, under the assumption that neutral diversity can be considered a proxy of effective population size. However, field studies that have compared quantitative genetic variation in populations of different size have not revealed any clear pattern (Willi et al., 2006). In our study, the correlation between functional color morph diversity and census population size was positive (not negative). Moreover, both functional diversity and census population size were larger (not smaller) in disturbed than in stable environments. These patterns cannot be accounted for by the potential response to selection or evolvability being greater in larger populations.

Our present analyses of genetic structure advance our understanding of the relative roles of selection, ecological events, and neutral processes as drivers of evolutionary divergence between populations of pygmy grasshoppers (Tinnert & Forsman, 2017; Tinnert, Hellgren et al., 2016) and other ecologically similar organisms. That neutral genetic differentiation between populations was associated with functional genetic differentiation (as revealed by Mantel test based on pairwise *F*_ST_ values for neutral and outlier AFLP loci) might indicate that divergence in functional traits has not only been influenced by selection, but also by gene flow and stochastic processes associated with founder events and drift in small populations (Dudaniec et al., 2018; Leinonen, McCairns, O'Hara, & Merila, 2013; Tibblin et al., 2015). However, pairwise population genetic differentiation was more pronounced on average for outlier loci than for neutral loci, and sampling location accounted for a greater proportion of the total variance in outlier (32%) than in neutral (8%) AFLP loci, thus implicating also natural selection and local adaptation as drivers of population divergence (Dudaniec et al., 2018; Johansson et al., 2016; Landguth & Balkenhol, 2012; Quintela et al., 2014).

That there was no genetic differentiation between populations in the two contrasting environments is an important finding in that it shows that stable and disturbed habitats were not populated by reproductively isolated evolutionary lineages or ecotypes. In our study, the disturbed sites were located on average at greater distances from the coast and at higher elevation than stable sites. However, two lines of evidence indicate that this geographic clustering did not confound the results and conclusions regarding the effects of disturbance on genetic diversity. First, there was no overall genetic structure or separation between populations in stable and disturbed environments. It is therefore unlikely that environmental conditions or ecological and evolutionary processes related to distance from the coast, but independent of the degree of disturbance, contributed in any important way to the genetic and phenotypic differences between stable and disturbed sites. Second, not only were populations that were closer to the coast genetically similar to inland populations, our estimates of neutral and functional genetic and phenotypic diversity within populations were independent of elevation. A plethora of factors can impact phenotypic, functional, and neutral genetic diversity of natural populations. The correlational approach of our study means that we cannot with certainty identify all the contributing and nonimportant mechanisms. However, our findings are in agreement with the notion that the relative importance of ecological and evolutionary processes as drivers of neutral and functional genetic diversity changes along the stability–disturbance gradient.

### 4.2 Extensions and caveats

Depending on the questions and hypothesis under investigation, contrasting environments can consist of habitats that represent different environmental conditions, disturbance regimes (as in the present study), populations inhabiting large (source) as opposed to small (sink) habitat patches, populations in ephemeral or temporal as opposed to permanent habitat patches, or populations at the core of the distribution range as opposed to populations in marginal areas that represent invasion fronts of expanding species (Johansson et al., 2016; Noguerales et al., 2016; Quintela et al., 2014; Shine, Brown, & Phillips, 2011; Sunde, Tamario, Tibblin, Larsson, & Forsman, 2018). To broaden utility, environments could be characterized not as binary states but along continuous gradients, such as age of habitat or time since disturbance event (Forsman, Karlsson et al., 2011).

Wing dimorphism is widespread among insects (Harrison, 1980; Schwander & Leimar, 2011). Our study provided more evidence that the incidence of long‐winged morph could thus be used as a proxy for recent establishment and immigration in many Orthoptera, Heteroptera, and Coleoptera species. In other animal groups, genetic expression of locomotion and dispersal‐enhancing quantitative phenotypic traits can be used as surrogates to infer recent establishment, recolonization, or immigration events (Arnold & Bennett, 1988; Berthouly‐Salazar, van Rensburg, Le Roux, van Vuuren, & Hui, 2012; Forsman, Merilä, & Ebenhard, 2011; Shine et al., 2011).

A benefit of using color morph diversity as a proxy for functionally important genetic and phenotypic variation in the present study is that color pattern of pygmy grasshoppers is genetically and developmentally associated with morphology, physiology, life history, and behaviors (Ahnesjö & Forsman, 2003, 2006; Forsman, 1999, 2018; Forsman et al., 2002, 2007, 2012 and references therein). Differences in color morph diversity between populations therefore likely reflect imprints of recent establishment and effects of selection acting directly on color pattern and on traits that are associated with color pattern, possibly resulting in a stronger signature. Color patterns covary with different functionally important phenotypic dimensions also in other groups of organisms (e.g., Mayr, 1963; McKinnon & Pierotti, 2010; McLean & Stuart‐Fox, 2014; True, 2003; Wellenreuther, Svensson, & Hanson, 2014), thus allowing for replication of the approach in other species. In species that are not color polymorphic, quantitative genetics estimates, such as genetic variances and narrow‐sense heritability (Roff, 1997; Walsh & Lynch, 2012), can be used to assess and compare functional genetic variation of phenotypic traits that influence individual fitness, evolutionary potential, and adaptability of populations (Leinonen et al., 2008, 2013; Reed & Frankham, 2001).

Developmental plasticity and phenotypic flexibility can severely impact variation in quantitative traits (Bradshaw, 1965; Forsman, 2015; West‐Eberhard, 2003) and potentially affect both the magnitude and direction of the difference in comparisons between neutral molecular and functional genetic diversity. This is not an issue in the present study because pygmy grasshopper color patterns and dispersal phenotypes are not affected to any important degree by developmental plasticity (Berggren et al., 2012; Forsman, 2018; Forsman, Karlsson et al., 2011; Karlsson et al., 2009; Nabours, 1929 and references therein). Available evidence also indicates that color morph and wing morph are independent in pygmy grasshoppers (Forsman, 2018).

Interpretation of results from comparisons based on estimates of quantitative genetic variation versus neutral variation is potentially complicated by the conversion of nonadditive (epistatic and dominance) to additive genetic variation and by the physical inhibition of recombination associated with chromosomal rearrangements in small populations (Neiman & Linksvayer, 2005; Walsh & Lynch, 2012; Willi et al., 2006). The increase in additive genetic variance resulting from these phenomena offers an alternative explanation to why functional phenotypic diversity may be higher than neutral diversity in recently established and bottlenecked populations, even in cases when functional diversity has not contributed to enhanced establishment or stabilized population dynamics. We cannot completely discard the possibility that such conversion of genetic variance has influenced the outcome of the comparison of color morph diversity in the present study. However, our finding that the difference between stable and disturbed environments in neutral AFLP diversity was opposite the diversity in outlier AFLP loci cannot be explained by conversion of nonadditive to additive genetic variation.

The color polymorphism in pygmy grasshoppers likely has a polygenic inheritance (Fisher, 1939; Nabours, 1929). A transcriptomic analysis on *Tetrix japonica*—a closely related pygmy grasshopper species (see Figure S1 in Forsman, 2018)—has identified several putative pigment‐related genes and differential expression patterns of them between adults and larval phases in this species (Qiu, Liu, Lu, & Huang, 2017). Moreover, putative genes involved in juvenile hormone metabolism and signaling pathways that regulate the body color and flight activity in insects are available for the same species (Qiu et al., 2017). A genomic approach taking advantage of advances in sequences technologies such as restriction site‐associated DNA sequencing (e.g., RADseq) and of the available genome‐wide sequencing data and protein expression profiles from related species can generate a more comprehensive understanding of the genes/loci under selection. More specifically, quantitative trait analyses can reveal the interplay between the loci that regulate polygenic traits (such as color patterns and wing morphs) to gain insights into the rapid evolution of relevant phenotypic traits by comparing DNA profiles from contrasting environments (Dudaniec et al., 2018; Johansson et al., 2016; Noguerales et al., 2016; Quintela et al., 2014; Zhi‐Xiang et al., 2018).

When interpreting results from comparisons of neutral and functional genetic diversity in populations between contrasting environments, it is necessary to take into consideration any systematic differences in effective population size. It is not only the eroding effect of genetic drift that depends on effective population size. Evolvability, or the efficiency by which selection can remove deleterious alleles and drive advantageous alleles to fixation, increases with increasing effective population size (Willi et al., 2006). If populations in disturbed, marginal, or temporal habitats are generally smaller than populations in stable, central, or continuous habitats, then this may contribute to a pattern of greater functional than neutral genetic diversity in the former type of environments. It is however unlikely that such a confounding effect of population size biased the results of the grasshopper population comparisons in the present study. If anything, study populations in disturbed environments were larger (not smaller) compared with populations in stable environments and yet they harbored higher functional compared to neutral diversity.

## 5 CONCLUSIONS

Our results for *T. undulata* grasshoppers illustrate that genetic diversity within natural populations is determined by a combination of ecological and evolutionary processes, the importance of which are different for neutral and functional genetic diversity and vary between environmental disturbance regimes (Figure 4). A higher proportion of the long‐winged phenotype in disturbed habitats suggested that these populations were founded by flight‐capable individuals shortly after the disturbance event. Both color morph diversity and outlier genetic dispersion were higher in disturbed than in stable habitats, a likely consequence of higher survival and establishment success of more color morph diverse founder groups (Forsman et al., 2007, 2012; Wennersten et al., 2012), combined with stabilizing selection reducing functional genetic variance in stable environments (Karlsson et al., 2008). Neutral genetic diversity was lower in populations that occupied disturbed habitats, as a result of recent colonization events and bottlenecks that reduce neutral genetic diversity. We also found that functional phenotypic color morph diversity was negatively correlated with neutral genetic diversity across populations, underscoring that diversity estimates based on neutral markers should not be used as substitutes for adaptive or functional genetic diversity to infer evolutionary potential and the ability of populations and species to cope with environmental change. Our findings highlight the utility of combining morphological data with outlier and neutral loci in analyzing the consequences of environmental change for population genetic structure and diversity, thus informing about key processes in ecology, evolution, and protection of biodiversity.

## CONFLICT OF INTEREST

None declared.

## AUTHOR CONTRIBUTIONS

A.F. designed the research. A.F. and J.T. collected animals. J.T. performed laboratory work. J.T. and Y.Y. analyzed molecular data. Y.Y., A.F., and J.T. performed statistical analyses. All authors interpreted the data and contributed to the writing of the paper.

## DATA ACCESSIBILITY

Data on sampling locations, sampling year, sex, and AFLP genotypes of individuals used for genetic analyses are available as electronic supplementary material.

## References

[ece34667-bib-0001] Ahnesjö, J. , & Forsman, A. (2003). Correlated evolution of colour pattern and body size in polymorphic pygmy grasshoppers, *Tetrix undulata* . Journal of Evolutionary Biology, 16, 1308–1318. 10.1046/j.1420-9101.2003.00610.x 14640422

[ece34667-bib-0002] Ahnesjö, J. , & Forsman, A. (2006). Differential habitat selection by pygmy grasshopper color morphs; interactive effects of temperature and predator avoidance. Evolutionary Ecology, 20, 235–257. 10.1007/s10682-006-6178-8

[ece34667-bib-0003] Anderson, M. J. (2001). A new method for non‐parametric multivariate analysis of variance. Austral Ecology, 26, 32–46.

[ece34667-bib-0004] Anderson, M. J. (2006). Distance‐based tests for homogeneity of multivariate dispersions. Biometrics, 62, 245–253. 10.1111/j.1541-0420.2005.00440.x 16542252

[ece34667-bib-0005] Anderson, M. J. , Gorley, R. N. , & Clarke, K. R. (2008). PERMANOVA+ for PRIMER: Guide to software and statistical methods. Plymouth, UK: PRIMER‐E.

[ece34667-bib-0006] Anderson, M. J. , & Walsh, D. C. I. (2013). PERMANOVA, ANOSIM, and the Mantel test in the face of heterogeneous dispersions: What null hypothesis are you testing? Ecological Monographs, 83, 557–574. 10.1890/12-2010.1

[ece34667-bib-0007] Arnold, S. J. , & Bennett, A. F. (1988). Behavioral variation in natural populations. V. Morphological correlates of locomotion in the garter snake *Thamnophis radix* . Biological Journal of the Linnean Society, 34, 175–190. 10.1111/j.1095-8312.1988.tb01955.x

[ece34667-bib-0008] Arnold, S. J. , & Wade, M. J. (1984). On the measurement of natural and sexual selection: Theory. Evolution, 38, 709–719. 10.1111/j.1558-5646.1984.tb00344.x 28555816

[ece34667-bib-0009] Benjamini, Y. , & Hochberg, Y. (1995). Controlling the false discovery rate: A practical and powerful approach to multiple testing. Journal of the Royal Statistical Society Series B (Methodological), 57, 289–300.

[ece34667-bib-0010] Bensch, S. , & Åkesson, M. (2005). Ten years of AFLP in ecology and evolution: Why so few animals. Molecular Ecology, 14, 2899–2914. 10.1111/j.1365-294X.2005.02655.x 16101761

[ece34667-bib-0011] Berggren, H. , Tinnert, J. , & Forsman, A. (2012). Spatial sorting may explain evolutionary dynamics of wing polymorphism in pygmy grasshoppers. Journal of Evolutionary Biology, 25, 2126–2138. https://doi.org/2110/1111/j.1420-9101.2012.02592.x 22901281 10.1111/j.1420-9101.2012.02592.x

[ece34667-bib-0012] Berthouly‐Salazar, C. , van Rensburg, B. J. , Le Roux, J. J. , van Vuuren, B. J. , & Hui, C. (2012). Spatial sorting drives morphological variation in the invasive bird, *Acridotheris tristis* . PLoS ONE, 7, e38145 10.1371/journal.pone.0038145 22693591 PMC3364963

[ece34667-bib-0013] Bolnick, D. I. , Amarasekare, P. , Araújo, M. S. , Bürger, R. , Levine, J. M. , Novak, M. , … Vasseur, D. A. (2011). Why intraspecific trait variation matters in community ecology. Trends in Ecology & Evolution, 26, 183–192. 10.1016/j.tree.2011.01.009 21367482 PMC3088364

[ece34667-bib-0014] Bonin, A. , Ehrich, D. , & Manel, S. (2007). Statistical analysis of amplified fragment length polymorphism data: A toolbox for molecular ecologists and evolutionists. Molecular Ecology, 16, 3737–3758. 10.1111/j.1365-294X.2007.03435.x 17850542

[ece34667-bib-0015] Bonnet, E. , & Van de Peer, Y. (2002). zt: A software tool for simple and partial Mantel tests. Journal of Statistical Software, 7, 1–12.

[ece34667-bib-0016] Bradshaw, A. D. (1965). Evolutionary significance of phenotypic plasticity in plants. Advances in Genetics, 13, 115–155.

[ece34667-bib-0017] Caesar, S. , Ahnesjö, J. , & Forsman, A. (2007). Testing the role of co‐adapted genes versus bet hedging for mating strategies in colour polymorphic pygmy grasshoppers. Biological Journal of the Linnean Society, 90, 491–499. 10.1111/(ISSN)1095-8312

[ece34667-bib-0018] Caesar, S. , Karlsson, M. , & Forsman, A. (2010). Diversity and relatedness enhance survival in colour polymorphic grasshoppers. PLoS ONE, 5, e10880 10.1371/journal.pone.0010880 20526364 PMC2878323

[ece34667-bib-0019] Charlesworth, B. , & Charlesworth, D. (2017). Population genetics from 1966 to 2016. Heredity, 118, 2–9. 10.1038/hdy.2016.55 27460498 PMC5176116

[ece34667-bib-0020] Chybicki, I. J. , Oleksa, A. , & Burczyk, J. (2011). Increased inbreeding and strong kinship structure in *Taxus baccata* estimated from both AFLP and SSR data. Heredity, 107, 589–600. 10.1038/hdy.2011.51 21712844 PMC3242636

[ece34667-bib-0021] Clarke, K. , & Gorley, R. (2006). PRIMER v6: User manual/tutorial. Plymouth, UK: PRIMER‐E Ltd.

[ece34667-bib-0022] Denno, R. E. , Roderick, G. K. , Peterson, M. A. , Huberty, A. F. , Dobel, H. G. , Eubanks, M. D. , … Langellotto, G. A. (1996). Habitat persistence underlies intraspecific variation in the dispersal strategies of planthoppers. Ecological Monographs, 66, 389–408. 10.2307/2963487

[ece34667-bib-0023] Des Roches, S. , Post, D. M. , Turley, N. E. , Bailey, J. K. , Hendry,A. P. , Kinnison,M. T. ,.. Palkovacs,E.P. . (2018). The ecological importance of intraspecific variation. Nature Ecology & Evolution, 2, 57–64. 10.1038/s41559-017-0402-5 29203921

[ece34667-bib-0104] de Vladar, H. P. , & Barton, N. (2014). Stability and response of polygenic traits to stabilizing selection and mutation. Genetics, 197, 749–767. 10.1534/genetics.113.159111 24709633 PMC4063930

[ece34667-bib-0024] Dudaniec, R. Y. , Yong, C. J. , Lancaster, L. T. , Svensson, E. I. , & Hansson, B. (2018). Signatures of local adaptation along environmental gradients in a range‐expanding damselfly (*Ischnura elegans*). Molecular Ecology, 27, 2576–2593. 10.1111/mec.14709 29707847

[ece34667-bib-0025] Endler, J. A. (1986). Natural selection in the wild. Princeton, NJ: Princeton University Press.

[ece34667-bib-0026] Ersts, P. (2012). Geographic distance matrix generator version 1.23. American Museum of Natural History, Center for Biodiversity and Conservation Available from: http://biodiversityinformatics.amnh.org/open_source/gdmg

[ece34667-bib-0027] Excoffier, L. , & Lischer, H. E. L. (2010). Arlequin suite ver 3.5: A new series of programs to perform population genetics analyses under Linux and Windows. Molecular Ecology Resources, 10, 564–567. 10.1111/j.1755-0998.2010.02847.x 21565059

[ece34667-bib-0028] Excoffier, L. , Smouse, P. E. , & Quattro, J. M. (1992). Analysis of molecular variance inferred from metric distances among DNA haplotypes: Application to human mitochondrial DNA restriction data. Genetics, 131, 479–491. 1644282 10.1093/genetics/131.2.479 PMC1205020

[ece34667-bib-0029] Fisher, R. A. (1939). Selective forces in wild populations of *Paratettix texanus* . Annals of Eugenics, 9, 109–122. 10.1111/j.1469-1809.1939.tb02201.x

[ece34667-bib-0030] Foll, M. , & Gaggiotti, O. (2008). A genome‐scan method to identify selected loci appropriate for both dominant and codominant markers: A Bayesian perspective. Genetics, 180, 977–993. 10.1534/genetics.108.092221 18780740 PMC2567396

[ece34667-bib-0031] Forsman, A. (1999). Reproductive life history variation among colour morphs of the pygmy grasshopper *Tetrix subulata* . Biological Journal of the Linnean Society, 67, 247–261. 10.1111/j.1095-8312.1999.tb01863.x

[ece34667-bib-0032] Forsman, A. (2011). Rethinking the thermal melanism hypothesis: Rearing temperature and coloration in pygmy grasshoppers. Evolutionary Ecology, 25, 1247–1257. 10.1007/s10682-011-9477-7

[ece34667-bib-0033] Forsman, A. (2014). Effects of genotypic and phenotypic variation on establishment are important for conservation, invasion and infection biology. Proceedings of the National Academy of Sciences USA, 111, 302–307. 10.1073/pnas.1317745111 PMC3890895 24367109

[ece34667-bib-0034] Forsman, A. (2015). Rethinking phenotypic plasticity and its consequences for individuals, populations and species. Heredity, 115, 276–284. 10.1038/hdy.2014.92 25293873 PMC4815454

[ece34667-bib-0035] Forsman, A. (2016). Is colour polymorphism advantageous to populations and species? Molecular Ecology, 25, 2693–2698. 10.1111/mec.13629 27178084

[ece34667-bib-0036] Forsman, A. (2018). On the role of sex differences for evolution in heterogeneous and changing fitness landscapes: Insights from pygmy grasshoppers. Philosophical Transactions of the Royal Society B: Biological Sciences, 373, 20170429 10.1098/rstb.2017.0429 PMC6125723 30150227

[ece34667-bib-0037] Forsman, A. , Ahnesjö, J. , & Caesar, S. (2007). Fitness benefits of diverse offspring in pygmy grasshoppers. Evolutionary Ecology Research, 9, 1305–1318.

[ece34667-bib-0038] Forsman, A. , Ahnesjö, J. , Caesar, S. , & Karlsson, M. (2008). A model of ecological and evolutionary consequences of color polymorphism. Ecology, 89, 34–40. 10.1890/07-0572.1 18376544

[ece34667-bib-0039] Forsman, A. , & Appelqvist, S. (1999). Experimental manipulation reveals differential effects of colour pattern on survival in male and female pygmy grasshoppers. Journal of Evolutionary Biology, 12, 391–401. 10.1046/j.1420-9101.1999.00041.x

[ece34667-bib-0040] Forsman, A. , Betzholtz, P. E. , & Franzén, M. (2015). Variable coloration is associated with dampened population fluctuations in noctuid moths. Proceedings of the Royal Society B, 282, 20142922 10.1098/rspb.2014.2922 25972462 PMC4455791

[ece34667-bib-0041] Forsman, A. , Betzholtz, P. E. , & Franzén, M. (2016). Faster poleward range shifts in moths with more variable colour patterns. Scientific Reports, 6, 36265 10.1038/srep36265 27808116 PMC5093557

[ece34667-bib-0042] Forsman, A. , Karlsson, M. , Wennersten, L. , Johansson, J. , & Karpestam, E. (2011). Rapid evolution of fire melanism in replicated populations of pygmy grasshoppers. Evolution, 65, 2530–2540. 10.1111/j.1558-5646.2011.01324.x 21884054

[ece34667-bib-0043] Forsman, A. , Merilä, J. , & Ebenhard, T. (2011). Phenotypic evolution of dispersal‐enhancing traits in insular voles. Proceedings of the Royal Society B: Biological Sciences, 278, 225–232. 10.1098/rspb.2010.1325 PMC3013397 20685710

[ece34667-bib-0044] Forsman, A. , Ringblom, K. , Civantos, E. , & Ahnesjö, J. (2002). Coevolution of color pattern and thermoregulatory behavior in polymorphic pygmy grasshoppers *Tetrix undulata* . Evolution, 56, 349–360. 10.1111/j.0014-3820.2002.tb01345.x 11926503

[ece34667-bib-0045] Forsman, A. , & Wennersten, L. (2016). Inter‐individual variation promotes ecological success of populations and species: Evidence from experimental and comparative studies. Ecography, 39, 630–648. 10.1111/ecog.01357

[ece34667-bib-0046] Forsman, A. , Wennersten, L. , Karlsson, M. , & Caesar, S. (2012). Variation in founder groups promotes establishment success in the wild. Proceedings of the Royal Society B, 279, 2800–2806. 10.1098/rspb.2012.0174 22456885 PMC3367781

[ece34667-bib-0047] Frankham, R. (1996). Relationship of genetic variation to population size in wildlife. Conservation Biology, 10, 1500–1508. 10.1046/j.1523-1739.1996.10061500.x

[ece34667-bib-0048] Harrison, R. G. (1980). Dispersal polymorphism in insects. Annual Review of Ecology and Systematics, 11, 95–118. 10.1146/annurev.es.11.110180.000523

[ece34667-bib-0049] Hedrick, P. W. (2006). Genetic polymorphism in heterogeneous environments: The age of genomics. Annual Review of Ecology and Systematics, 37, 67–93. 10.1146/annurev.ecolsys.37.091305.110132

[ece34667-bib-0050] Holderegger, R. , Kamm, U. , & Gugerli, F. (2006). Adaptive vs. neutral genetic diversity: Implications for landscape genetics. Landscape Ecology, 21, 797–807. 10.1007/s10980-005-5245-9

[ece34667-bib-0051] Holst, K. T. (1986). The Saltatoria of Northern Europe. Fauna Entomologica Scandinavica, 16, 1–127.

[ece34667-bib-0052] Hughes, A. R. , Inouye, B. D. , Johnson, M. T. J. , Underwood, N. , & Vellend, M. (2008). Ecological consequences of genetic diversity. Ecology Letters, 11, 609–623. 10.1111/j.1461-0248.2008.01179.x 18400018

[ece34667-bib-0053] Jeffrey, H. (1961). Theory of probability (3rd ed.). Oxford, UK: Oxford University Press.

[ece34667-bib-0054] Johansson, J. , Caesar, S. , & Forsman, A. (2013). Multiple paternity increases phenotypic diversity in *Tetrix subulata* pygmy grasshoppers. Journal of Orthoptera Research, 22, 79–85. 10.1665/034.022.0204

[ece34667-bib-0055] Johansson, M. P. , Quintela, M. , & Laurila, A. (2016). Genetic divergence and isolation by thermal environment in geothermal populations of an aquatic invertebrate. Journal of Evolutionary Biology, 29, 1701–1712. 10.1111/jeb.12902 27208484

[ece34667-bib-0056] Kader, G. , & Perry, M. (2007). Variability for categorical variables. Journal of Statistics Education, 15 http://www.amstat.org/publications/jse/v15n12/kader.pdf

[ece34667-bib-0057] Karlsson, M. , Caesar, S. , Ahnesjö, J. , & Forsman, A. (2008). Dynamics of colour polymorphism in changing environments: Fire melanism and then what? Oecologia, 154, 715–724. 10.1007/s00442-007-0876-y 17957385

[ece34667-bib-0058] Karlsson, M. , & Forsman, A. (2010). Is melanism in pygmy grasshoppers induced by crowding? Evolutionary Ecology, 24, 975–983. 10.1007/s10682-010-9399-9

[ece34667-bib-0059] Karlsson, M. , Johansson, J. , Caesar, S. , & Forsman, A. (2009). No evidence for developmental plasticity of color patterns in response to rearing substrate in pygmy grasshoppers. Canadian Journal of Zoology‐Revue Canadienne De Zoologie, 87, 1044–1051. 10.1139/Z09-097

[ece34667-bib-0060] Karpestam, E. , Merilaita, S. , & Forsman, A. (2013). Detection experiments with humans implicate visual predation as a driver of colour polymorphism dynamics in pygmy grasshoppers. BMC Ecology, 13, 17 10.1186/1472-6785-13-17 23639215 PMC3648452

[ece34667-bib-0061] Karpestam, E. , Merilaita, S. , & Forsman, A. (2014). Body size influences differently the detectabilities of colour morphs of cryptic prey. Biological Journal of the Linnean Society, 113, 112–122. 10.1111/bij.12291

[ece34667-bib-0062] Karpestam, E. , Merilaita, S. , & Forsman, A. (2016). Colour polymorphism protects prey individuals and populations against predation. Scientific Reports, 6, 22122 10.1038/srep22122 26902799 PMC4763262

[ece34667-bib-0063] Kimura, M. (1983). The neutral theory of molecular evolution In Nei M. & Koehn R. K. (Eds.), Evolution of genes and proteins. Sunderland, MA: Sinauer Associates.

[ece34667-bib-0064] Kruskal, J. B. (1964). Nonmetric multidimensional scaling: A numerical method. Psychometrika, 29, 115–129. 10.1007/BF02289694

[ece34667-bib-0065] Landguth, E. L. , & Balkenhol, N. (2012). Relative sensitivity of neutral versus adaptive genetic data for assessing population differentiation. Conservation Genetics, 13, 1421–1426. 10.1007/s10592-012-0354-x

[ece34667-bib-0066] Leinonen, T. , McCairns, R. J. S. , O'Hara, R. B. , & Merila, J. (2013). QST‐FST comparisons: Evolutionary and ecological insights from genomic heterogeneity. Nature Review of Genetics, 14, 179–190. 10.1038/nrg3395 23381120

[ece34667-bib-0067] Leinonen, T. , O'Hara, R. B. , Cano, J. M. , & Merilä, J. (2008). Comparative studies of quantitative trait and neutral marker divergence: A meta‐analysis. Journal of Evolutionary Biology, 21, 1–17. 10.1111/j.1420-9101.2007.01445.x 18028355

[ece34667-bib-0068] Lenormand, T. (2002). Gene flow and the limits to natural selection. Trends in Ecology & Evolution, 17, 183–189. 10.1016/S0169-5347(02)02497-7

[ece34667-bib-0069] Mayr, E. (1963). Animal species and evolution. Cambridge, MA: Harvard University Press 10.4159/harvard.9780674865327

[ece34667-bib-0070] McArdle, B. H. , & Anderson, M. J. (2001). Fitting multivariate models to community data: A comment on distance‐based redundancy analysis. Ecology, 82, 290–297. 10.1890/0012-9658(2001)082%5b0290:FMMTCD%5d2.0.CO;2

[ece34667-bib-0071] McKinnon, J. S. , & Pierotti, M. E. R. (2010). Colour polymorphism and correlated characters: Genetic mechanisms and evolution. Molecular Ecology, 19, 5101–5125. 10.1111/j.1365-294X.2010.04846.x 21040047

[ece34667-bib-0072] McLean, C. A. , & Stuart‐Fox, D. (2014). Geographic variation in animal colour polymorphisms and its role in speciation. Biological Reviews, 89, 860–873. 10.1111/brv.12083 24528520

[ece34667-bib-0073] Mills, L. S. , Bragina, E. V. , Kumar, A. V. , Zimova, M. , Lafferty, D. J. R. , Feltner, J. , … Fay, K. (2018). Winter color polymorphisms identify global hot spots for evolutionary rescue from climate change. Science, 359, 1033–1036. 10.1126/science.aan8097 29449510

[ece34667-bib-0074] Nabours, R. K. (1929). The genetics of the Tettigidae. Bibliographia Genetica, 5, 27–104.

[ece34667-bib-0075] Nei, M. (1973). Analysis of gene diversity in subdivided populations. Proceedings of the National Academy of Sciences, 70, 3321–3323. 10.1073/pnas.70.12.3321 PMC427228 4519626

[ece34667-bib-0076] Neiman, M. , & Linksvayer, T. A. (2005). The conversion of variance and the evolutionary potential of restricted recombination. Heredity, 96, 111–121. 10.1038/sj.hdy.6800772 16333302

[ece34667-bib-0077] Noguerales, V. , García‐Navas, V. , Cordero, P. J. , & Ortego, J. (2016). The role of environment and core‐margin effects on range‐wide phenotypic variation in a montane grasshopper. Journal of Evolutionary Biology, 29, 2129–2142. 10.1111/jeb.12915 27271999

[ece34667-bib-0078] Parker, K. A. , Anderson, M. J. , Jenkins, P. F. , & Brunton, D. H. (2012). The effects of translocation‐induced isolation and fragmentation on the cultural evolution of bird song. Ecology Letters, 15, 778–785. 10.1111/j.1461-0248.2012.01797.x 22590997

[ece34667-bib-0079] Perry, M. , & Kader, G. (2005). Variation as unalikeability. Teaching Statistics, 27, 58–60. 10.1111/j.1467-9639.2005.00210.x

[ece34667-bib-0080] Qiu, Z. , Liu, F. , Lu, H. , & Huang, Y. (2017). Characterization and analysis of a de novo transcriptome from the pygmy grasshopper *Tetrix japonica* . Molecular Ecology Resources, 17, 381–392. 10.1111/1755-0998.12553 27288670

[ece34667-bib-0081] Quintela, M. , Johansson, M. P. , Kristjansson, B. K. , Barreiro, R. , & Laurila, A. (2014). AFLPs and mitochondrial haplotypes reveal local adaptation to extreme thermal environments in a freshwater gastropod. PLoS ONE, 9, e101821 10.1371/journal.pone.0101821 25007329 PMC4090234

[ece34667-bib-0082] Reed, D. H. , & Frankham, R. (2001). How closely correlated are molecular and quantitative measures of genetic variation? A meta‐analysis. Evolution, 55, 1095–1103. 10.1111/j.0014-3820.2001.tb00629.x 11475045

[ece34667-bib-0083] Reed, D. H. , & Frankham, R. (2003). Correlation between fitness and genetic diversity. Conservation Biology, 17, 230–237. 10.1046/j.1523-1739.2003.01236.x

[ece34667-bib-0084] Rius, M. , & Darling, J. A. (2014). How important is intraspecific genetic admixture to the success of colonising populations? Trends in Ecology & Evolution, 29, 233–242. 10.1016/j.tree.2014.02.003 24636862

[ece34667-bib-0085] Roff, D. A. (1997). Evolutionary quantitative genetics. New York, NY: Chapman & Hall 10.1007/978-1-4615-4080-9

[ece34667-bib-0086] Roff, D. A. , & Fairbairn, D. J. (2007). The evolution and genetics of migration in insects. BioScience, 57, 155–164. 10.1641/B570210

[ece34667-bib-0087] Sambrook, J. , Fritch, F. J. , & Maniatis, T. (2002). Molecular cloning, a laboratory manual. Cold Spring Harbor, NY: Cold Spring Harbor Laboratory Press.

[ece34667-bib-0088] Schluter, P. M. , & Harris, S. A. (2006). Analysis of multilocus fingerprinting data sets containing missing data. Molecular Ecology Notes, 6, 569–572. 10.1111/j.1471-8286.2006.01225.x

[ece34667-bib-0089] Schwander, T. , & Leimar, O. (2011). Genes as leaders and followers in evolution. Trends in Ecology and Evolution, 26, 143–151. 10.1016/j.tree.2010.12.010 21257223

[ece34667-bib-0090] Shine, R. , Brown, G. P. , & Phillips, B. L. (2011). An evolutionary process that assembles phenotypes through space rather than through time. Proceedings of the National Academy of Sciences USA, 108, 5708–5711. 10.1073/pnas.1018989108 PMC3078378 21436040

[ece34667-bib-0091] Simberloff, D. (2009). The role of propagule pressure in biological invasions. Annual Review of Ecology and Systematics, 40, 81–102. 10.1146/annurev.ecolsys.110308.120304

[ece34667-bib-0092] Slatkin, M. (1987). Gene flow and the geographic structure of natural‐populations. Science, 236, 787–792. 10.1126/science.3576198 3576198

[ece34667-bib-0093] Slatkin, M. (1993). Isolation by distance in equilibrium and nonequilibrium populations. Evolution, 47, 264–279. 10.1111/j.1558-5646.1993.tb01215.x 28568097

[ece34667-bib-0094] Slatkin, M. (2008). Linkage disequilibrium–understanding the evolutionary past and mapping the medical future. Nature Reviews Genetics, 9, 477–485. 10.1038/nrg2361 PMC5124487 18427557

[ece34667-bib-0095] Sunde, J. , Tamario, C. , Tibblin, P. , Larsson, P. , & Forsman, A. (2018). Variation in salinity tolerance between and within anadromous subpopulations of pike (*Esox lucius)* . Scientific Reports, 8, 22 10.1038/s41598-017-18413-8 29311634 PMC5758576

[ece34667-bib-0096] Tibblin, P. , Forsman, A. , Koch‐Schmidt, P. , Nordahl, O. , Johannessen, P. , Nilsson, J. , & Larsson, P. (2015). Evolutionary divergence of adult body size and juvenile growth in sympatric subpopulations of a top predator in aquatic ecosystems. The American Naturalist, 186, 98–110. 10.1086/681597 26098342

[ece34667-bib-0097] Tinnert, J. , Berggren, H. , & Forsman, A. (2016). Population‐specific effects of interbreeding and admixture on reproductive decisions and offspring quality. Annales Zoologici Fennici, 53, 55–68. 10.5735/086.053.0205

[ece34667-bib-0098] Tinnert, J. , & Forsman, A. (2017). The role of dispersal for genetic and phenotypic variation: Insights from comparisons of sympatric pygmy grasshoppers. Biological Journal of the Linnean Society, blx055, 84–97. 10.1093/biolinnean/blx055

[ece34667-bib-0099] Tinnert, J. , Hellgren, O. , Lindberg, J. , Koch‐Schmidt, P. , & Forsman, A. (2016). Population genetic structure, differentiation and diversity in *Tetrix subulata* pygmy grasshoppers: Roles of population size and immigration. Ecology and Evolution, 6, 7831–7846. 10.1002/ece3.2520 30128133 PMC6093165

[ece34667-bib-0100] True, J. R. (2003). Insect melanism: The molecules matter. Trends in Ecology and Evolution, 18, 640–647. 10.1016/j.tree.2003.09.006

[ece34667-bib-0101] Tsurui, K. , Honma, A. , & Nishida, T. (2010). Camouflage effects of various colour‐marking morphs against different microhabitat backgrounds in a polymorphic pygmy grasshopper *Tetrix japonica* . PLoS ONE, 5(7), e11446 10.1371/journal.pone.0011446 20625405 PMC2897885

[ece34667-bib-0102] Vekemans, X. (2002). AFLP‐surv version 1.0. Distributed by the author. Brussels, Belgium: Laboratoire de Génetique et Ecologie Végétale, Univesité Libre de Bruxelles.

[ece34667-bib-0103] Vergeer, P. , Sonderen, E. , & Ouborg, N. J. (2004). Introduction strategies put to the test: Local adaptation versus heterosis. Conservation Biology, 18, 812–821. 10.1111/j.1523-1739.2004.00562.x

[ece34667-bib-0105] Vos, P. , Hogers, R. , Bleeker, M. , et al. (1995). AFLP: A new technique for DNA fingerprinting. Nucleic Acids Research, 23, 4407–4414. 10.1093/nar/23.21.4407 7501463 PMC307397

[ece34667-bib-0106] Walsh, B. , & Lynch, M. (2012). Evolution and Selection of Quantitative Traits. Online version.

[ece34667-bib-0107] Weir, B. S. , & Cockerham, C. C. (1984). Estimating F‐statistics for the analysis of population structure. Evolution, 38, 1358–1370. 28563791 10.1111/j.1558-5646.1984.tb05657.x

[ece34667-bib-0108] Wellenreuther, M. , Svensson, E. I. , & Hanson, B. (2014). Sexual selection and genetic colour polymorphisms in animals. Molecular Ecology, 23, 5398–5414. 10.1111/mec.12935 25251393

[ece34667-bib-0109] Wennersten, L. , & Forsman, A. (2012). Population‐level consequences of polymorphism, plasticity and randomized phenotype switching: A review of predictions. Biological Reviews, 87, 756–767. 10.1111/j.1469-185X.2012.00231.x 22540928

[ece34667-bib-0110] Wennersten, L. , Johansson, J. , Karpestam, E. , & Forsman, A. (2012). Higher establishment success in more diverse groups of pygmy grasshoppers under seminatural conditions. Ecology, 93, 2519–2525. 10.1890/12-0550.1 23431583

[ece34667-bib-0111] West‐Eberhard, M. J. (2003). Developmental plasticity and evolution. Oxford, UK: Oxford University Press.

[ece34667-bib-0112] Whitlock, R. (2014). Relationships between adaptive and neutral genetic diversity and ecological structure and functioning: A meta‐analysis. Journal of Ecology, 102, 857–872. 10.1111/1365-2745.12240 25210204 PMC4142011

[ece34667-bib-0113] Whitlock, R. , Hipperson, H. , Mannarelli, M. , Butlin, R. K. , & Burke, T. (2008). An objective, rapid and reproducible method for scoring AFLP peak‐height data that minimizes genotyping error. Molecular Ecology Resources, 8, 725–735. 10.1111/j.1755-0998.2007.02073.x 21585880

[ece34667-bib-0114] Willi, Y. , Van Buskirk, J. , & Hoffmann, A. A. (2006). Limits to the adaptive potential of small populations. Annual Review of Ecology and Systematics, 37, 433–458. 10.1146/annurev.ecolsys.37.091305.110145

[ece34667-bib-0115] Wright, S. (1943). Isolation by distance. Genetics, 28, 114–138. 17247074 10.1093/genetics/28.2.114 PMC1209196

[ece34667-bib-0116] Zhivotovsky, L. A. (1999). Estimating population structure in diploids with multilocus dominant DNA markers. Molecular Ecology, 8, 907–913. 10.1046/j.1365-294x.1999.00620.x 10434412

[ece34667-bib-0117] Zhi‐Xiang, P. , Fang, H. , & Guo‐Fang, J. (2018). Morphometrics reveal correlation between morphology and bioclimatic factors and population mixture in *Tetrix japonica* (Orthoptera: Tetrigidae). Acta Zoologica, 99, 199–210.

