# Supplementary material for: Contrasting patterns of neutral and functional genetic diversity in stable and disturbed environments
Source: Ecol Evol. 2018 Nov 11;8(23):12073–89. doi: 10.1002/ece3.4667 (PMC6303714; doi:10.1002/ece3.4667)
Supplement: Supplementary file 1 [file ECE3-8-12073-s001.docx]

**Contrasting patterns of neutral and functional genetic diversity in stable and disturbed environments**

Yeserin Yildirim, Jon Tinnert and Anders Forsman^*^

**Supporting Information**

**Supporting information Methods**

***DNA extraction and molecular genetics analyses***

From each of the 20 *Tetrix undulata* pygmy grasshopper sampling locations we used 5 – 28 individuals for DNA extraction and genetic analysis (**Table 1**). DNA was extracted from the femur of each individual. The femur was put in 500μl SET-buffer (0.015M NaCl, 0.05M Tris, 0.001M EDTA, pH 8.0) and homogenized on a Qiagen Tissulyser at 23rps for 3 min. We added 7.5 μl Proteinase K (20mg/ml)) and 13μl SDS (20%) to each sample and kept them in a water bath (55°C) over night. The samples were then extracted using Phenol-Chloroform method according to Sambrook (Sambrook *et al.* 2002). The extracted DNA was diluted in 25μl 1XTE buffer, and quantified using a Nanodrop 2000 (Thermo Scientific). Each sample was diluted to a final concentration of 25ng/μl.

Analysis of amplified fragment length polymorphism (AFLP) was carried out as described by Vos (Vos *et al.* 1995; Bensch & Åkesson 2005), using the restriction enzymes EcoRI and Tru1, the pre amplification primers M_A_ X E_A_ and combinations of four selective primers (pair 1-E_TAG_ X M_CGA_, pair 2-E_TAG_ X M_CAG_, pair 3-E_TCG_ X M_CAC_, pair 4-E_TAG_ X M_CAC_). Three negative and nine positive controls were included on each plate. PCR products were diluted 8-fold, and 2μl of the dilutions were sent to Uppsala Genome Center for fragment analysis using capillary electrophoresis on an ABI3730XL DNA Analyzer (Applies Biosystems). Chromatograms from all PCR-plates were visually evaluated using GeneMarker 2.6.4 (SoftGenetics), and twelve PCR-plates were judged to be of lower chromatogram signal quality and subsequently resent for a second fragment analysis with a higher DNA concentration, thus reducing the risk of introducing error variance from plate effects. Individual chromatogram quality was assessed using GeneMarker 2.6.4 (SoftGenetics) and cleaned by removing chromatograms with poor quality. A second complementary analysis of AFLP was conducted for primer pairs lacking in the dataset, thus adding individuals otherwise removed by lack of data. Chromatograms were visualized and allele sizing and calling were performed in GeneMapper 5.0 (Applied Biosystems). A total of 1419 polymorphic sites were used for further analysis. Peak heights were normalized using AFLPscore (Whitlock *et al.* 2008), and were scored as present if the peaks were higher than 15% of the mean peak height of the particular locus. The fragments considered for scoring had a size range of 50-500 bp with a threshold fluorescence value of 200 arbitrary units. However, the fragments <125 bp were removed from the analyses to decrease the size homoplasy after preliminary analyses with Principal Component Analysis showed their removal resulted in only a negligible change in overall pattern (data not shown). The resulting binary matrix, consisting of ones and zeros depending on presence or absence of the fragment, was exported to Microsoft Excel for subsequent error rate calculation. Nine individuals, all from different collection sites, were replicated across all plates to measure genotyping repeatability, which was calculated for each allele using mismatch error rate (Bonin *et al.* 2004). The average error rate found across all the loci was 4.8% (range 1.1% – 14%). All DNA extraction were performed in the same laboratory (Lund University, Lund) and our results and conclusions are therefore not influenced by any difficulties associated with transferring AFLP information across laboratories.

SUPPORTING REFERENCES

Bensch S, Åkesson M (2005) Ten years of AFLP in ecology and evolution: why so few animals. *Molecular Ecology* **14**, 2899-2914.

Bonin A, Bellemain E, Eidesen PB*, et al.* (2004) How to track and assess genotyping errors in population genetics studies. *Molecular Ecology* **13**, 3261-3273.

Sambrook J, Fritch FJ, Maniatis T (2002) *Molecular clooning, a labaratory manual* Cold Spring Harbor Laboratory Press, Cold Spring Harbor, NY.

Whitlock R, Hipperson H, Mannarelli M, Butlin RK, Burke T (2008) An objective, rapid and reproducible method for scoring AFLP peak-height data that minimizes genotyping error. *Molecular Ecology Resources* **8**, 725-735.

Vos P, Hogers R, Bleeker M*, et al.* (1995) AFLP: a new technique for DNA fingerprinting. *Nucleic Acids Research* **23**, 4407-4414.

**Supporting information Table S1.** AMOVA results on *F_ST_*-statistics for *Tetrix undulata* pygmy grasshoppers. The hierarchical distribution of variation was determined between the sampling locations without introducing any structure (results below the dashed lines), and nesting the 20 populations in two different environments (disturbed and undisturbed, results above the dashed lines). AMOVA was performed for 1208 neutral loci (nDATA), and 28 outlier loci (sDATA).

|  | **Source of variation** | **d.f.** | **Sum of squares** | **Variance components** | **% of total variation** | **Fixation indexes** | ***P*** |
| --- | --- | --- | --- | --- | --- | --- | --- |
| **Neutral loci (nDATA)** | Between environments | 1 | 446.248 | 0.72080 | 0.41 | *F_CT_*: 0.00412 | 0.162±0.004 |
|  | Among populations within environments | 18 | 6824.993 | 13.03376 | 7.45 | *F_SC_*: 0.07481 | <0.001 |
|  | Within populations | 315 | 50773.482 | 161.18566 | 92.14 | *F_ST_*: 0.07862 | <0.001 |
|  | Total | 334 | 58044.722 | 174.94021 |  |  |  |
|  | Among populations | 19 | 7271.241 | 13.32778 | 7.64 | *F_ST_*: 0.07637 | <0.001 |
|  | Within populations | 315 | 50773.482 | 161.18566 | 92.36 |  | <0.001 |
|  | Total | 334 | 58044.722 | 174.51343 |  |  |  |
| **Outliers (sDATA)** | Between environments | 1 | 56.666 | 0.18488 | 2.86 | *F_ST_*: 0.02857 | 0.126±0.003 |
|  | Among populations within environments | 18 | 660.071 | 1.93229 | 29.86 | *F_SC_*: 0.30736 | <0.001 |
|  | Within populations | 315 | 1371.657 | 4.35447 | 67.29 | *F_ST_*: 0.32715 | <0.001 |
|  | Total | 334 | 2088.394 | 6.47164 |  |  |  |
|  | Among populations | 19 | 716.737 | 2.00770 | 31.56 | *F_ST_*: 0.31557 | <0.001 |
|  | Within populations | 315 | 1371.657 | 4.35447 | 68.44 |  | <0.001 |
|  | Total | 334 | 2088.394 | 6.36217 |  |  |  |

**Supporting information Table S2.** Pairwise *F_ST_* values across 20 populations of *Tetrix undulata* pygmy grasshoppers based on 28 outlier AFLP loci (above diagonal) and 1208 neutral AFLP loci (below the diagonal). Values in bold are significant (*P* < 0.05) after FDR correction based on 10,000 permutations.

|  | A22 | A27 | A30 | A31 | A32 | A33 | A39 | A40 | A42 | A43 | A46 | A47 | A48 | A51 | A53 | A55 | A57 | A58 | A61 | A62 |
| --- | --- | --- | --- | --- | --- | --- | --- | --- | --- | --- | --- | --- | --- | --- | --- | --- | --- | --- | --- | --- |
| A22 |  | **0.244** | **0.140** | **0.235** | **0.248** | **0.283** | **0.295** | **0.379** | **0.332** | **0.261** | **0.277** | **0.482** | **0.448** | **0.343** | **0.390** | **0.459** | **0.293** | **0.439** | **0.404** | **0.382** |
| A27 | **0.064** |  | **0.217** | **0.334** | **0.296** | **0.280** | **0.205** | **0.328** | **0.279** | **0.305** | **0.227** | **0.470** | **0.407** | **0.345** | **0.330** | **0.410** | **0.356** | **0.414** | **0.416** | **0.430** |
| A30 | **0.062** | **0.064** |  | 0.037 | **0.125** | **0.152** | **0.214** | **0.328** | **0.273** | **0.218** | **0.248** | **0.506** | **0.484** | **0.375** | **0.436** | **0.486** | **0.358** | **0.432** | **0.440** | **0.453** |
| A31 | **0.086** | **0.138** | **0.054** |  | -0.033 | 0.100 | **0.206** | **0.272** | **0.240** | **0.131** | **0.222** | **0.531** | **0.514** | **0.354** | **0.446** | **0.506** | **0.445** | **0.429** | **0.496** | **0.575** |
| A32 | **0.073** | **0.111** | **0.048** | -0.006 |  | **0.087** | **0.135** | **0.167** | **0.148** | **0.046** | **0.185** | **0.420** | **0.420** | **0.280** | **0.365** | **0.404** | **0.371** | **0.326** | **0.396** | **0.477** |
| A33 | **0.059** | **0.092** | **0.044** | 0.027 | 0.015 |  | **0.088** | **0.120** | **0.065** | **0.090** | **0.154** | **0.503** | **0.487** | **0.345** | **0.419** | **0.466** | **0.478** | **0.441** | **0.476** | **0.574** |
| A39 | **0.089** | **0.133** | **0.086** | **0.034** | **0.022** | 0.006 |  | 0.033 | **0.049** | **0.089** | **0.191** | **0.475** | **0.437** | **0.304** | **0.355** | **0.402** | **0.433** | **0.390** | **0.467** | **0.529** |
| A40 | **0.114** | **0.155** | **0.103** | **0.052** | **0.028** | **0.029** | **0.019** |  | 0.027 | **0.056** | **0.195** | **0.452** | **0.443** | **0.294** | **0.371** | **0.392** | **0.499** | **0.380** | **0.470** | **0.566** |
| A42 | **0.093** | **0.109** | **0.089** | **0.062** | **0.041** | 0.006 | 0.014 | **0.028** |  | **0.064** | **0.141** | **0.448** | **0.419** | **0.281** | **0.348** | **0.387** | **0.479** | **0.387** | **0.454** | **0.549** |
| A43 | **0.104** | **0.153** | **0.084** | 0.022 | 0.008 | **0.041** | **0.035** | **0.022** | **0.054** |  | **0.186** | **0.384** | **0.403** | **0.239** | **0.341** | **0.362** | **0.407** | **0.302** | **0.372** | **0.484** |
| A46 | **0.076** | **0.081** | **0.069** | **0.087** | **0.062** | **0.025** | **0.052** | **0.073** | **0.032** | **0.092** |  | **0.249** | **0.211** | **0.153** | **0.171** | **0.214** | **0.393** | **0.233** | **0.242** | **0.462** |
| A47 | **0.104** | **0.157** | **0.108** | **0.066** | **0.037** | **0.074** | **0.064** | **0.054** | **0.078** | **0.035** | **0.095** |  | 0.040 | 0.031 | **0.080** | 0.022 | **0.464** | 0.025 | **0.217** | **0.519** |
| A48 | **0.078** | **0.103** | **0.084** | **0.049** | **0.043** | **0.037** | **0.051** | **0.069** | **0.057** | **0.059** | **0.050** | **0.022** |  | **0.062** | 0.009 | 0.039 | **0.455** | **0.138** | **0.315** | **0.514** |
| A51 | **0.065** | **0.108** | **0.073** | **0.036** | **0.025** | **0.029** | **0.037** | **0.040** | **0.046** | **0.031** | **0.049** | -0.001 | 0.007 |  | **0.046** | 0.029 | **0.359** | 0.010 | **0.171** | **0.408** |
| A53 | **0.058** | **0.082** | **0.073** | **0.051** | **0.046** | **0.031** | **0.043** | **0.066** | **0.053** | **0.062** | **0.033** | **0.035** | -0.002 | 0.010 |  | 0.016 | **0.376** | **0.103** | **0.224** | **0.426** |
| A55 | **0.077** | **0.114** | **0.077** | **0.048** | **0.034** | **0.036** | **0.041** | **0.041** | **0.053** | **0.035** | **0.053** | 0.003 | 0.006 | -0.002 | 0.009 |  | **0.449** | **0.084** | **0.226** | **0.493** |
| A57 | **0.077** | **0.149** | **0.097** | **0.126** | **0.108** | **0.129** | **0.155** | **0.183** | **0.170** | **0.132** | **0.154** | **0.145** | **0.125** | **0.116** | **0.110** | **0.126** |  | **0.395** | **0.340** | 0.042 |
| A58 | **0.142** | **0.206** | **0.118** | **0.072** | **0.055** | **0.109** | **0.095** | **0.093** | **0.137** | **0.037** | **0.157** | **0.034** | **0.073** | **0.052** | **0.082** | **0.051** | **0.134** |  | **0.190** | **0.462** |
| A61 | **0.081** | **0.194** | **0.106** | **0.134** | **0.094** | **0.130** | **0.156** | **0.158** | **0.173** | **0.110** | **0.173** | **0.110** | **0.136** | **0.093** | **0.121** | **0.100** | **0.062** | **0.099** |  | **0.344** |
| A62 | **0.098** | **0.165** | **0.117** | **0.148** | **0.124** | **0.158** | **0.179** | **0.203** | **0.197** | **0.153** | **0.173** | **0.156** | **0.129** | **0.130** | **0.117** | **0.134** | **0.006** | **0.148** | **0.105** |  |
